# Tracking Charge Dynamics in a Silver Single-Atom Catalyst During the Light-Driven Oxidation of Benzyl Alcohol to Benzaldehyde

**DOI:** 10.1021/acscatal.4c05208

**Published:** 2025-03-21

**Authors:** Areti Moutsiou, Andrea Olivati, Luis A. Cipriano, Alessandra Sivo, Sean M. Collins, Quentin M. Ramasse, Ik Seon Kwon, Giovanni Di Liberto, Mohamad Kanso, Robert Wojcieszak, Gianfranco Pacchioni, Annamaria Petrozza, Gianvito Vilé

**Affiliations:** † Department of Chemistry, Materials, and Chemical Engineering “Giulio Natta”, Politecnico di Milano, Piazza Leonardo da Vinci 32, 20133 Milano, Italy; ‡ Center for Nanoscience and Technology, Italian Institute of Technology, Via Giovanni Pascoli 70/3, 20133 Milano, Italy; § Physics Department, Politecnico di Milano, Piazza Leonardo da Vinci 32, 20133 Milano, Italy; ∥ Bragg Centre for Materials Research, School of Chemical and Process Engineering and School of Chemistry, University of Leeds, Woodhouse Lane, LS2 9JT Leeds, U.K.; ⊥ SuperSTEM Laboratory, SciTech Daresbury Campus, Keckwick Lane, WA4 4AD Daresbury, U.K.; # School of Chemical and Process Engineering and School of Physics, University of Leeds, Woodhouse Lane, LS2 9JT Leeds, U.K.; ¶ Department of Energy Science & Engineering, Kunsan National University, 558 Daehak-ro, 54150 Gunsan, Republic of Korea; ∇ Department of Materials Science, University of Milan Bicocca, Via Roberto Cozzi 55, 20125 Milano, Italy; ○ Centre National de la Recherche Scientifique (CNRS) and Laboratoire Lorraine de Chimie Moléculaire, L2CM UMR 7053, Université de Lorraine, 54500 Vandœuvre-lès-Nancy, France

**Keywords:** single-atom catalyst, benzyl alcohol oxidation, density functional theory, transient absorption spectroscopy, photocatalysis

## Abstract

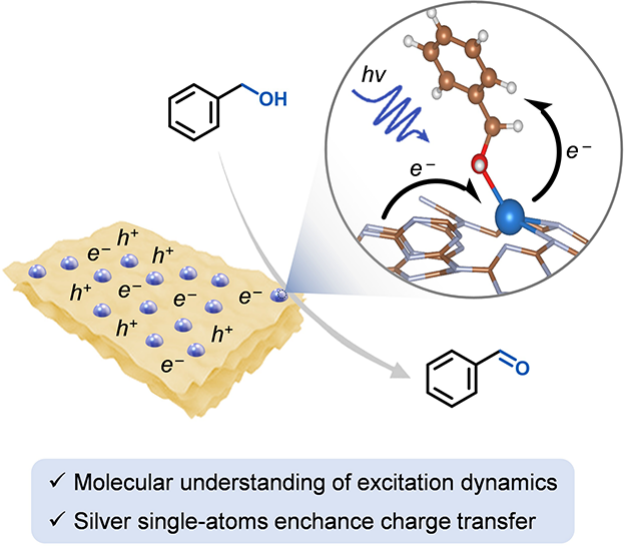

Understanding charge transfer in light-driven processes is crucial for optimizing the efficiency and performance of a photocatalyst, as charge transfer directly influences the separation and migration of photogenerated charge carriers and determines the overall reaction rate and product formation. However, achieving this understanding remains challenging in the context of single-atom photocatalysis. This study addresses this gap and investigates an Ag-based single-atom catalyst (Ag_1_@CN_*x*_) in the photocatalytic oxidation of benzyl alcohol to benzaldehyde. Comprehensive characterization was conducted using a battery of diffractive, textural, spectroscopic, and microscopic methods, confirming the catalyst crystallinity, porosity, elemental composition, and atomic dispersion of silver atoms. This material displayed efficient performance in the selective oxidation of benzyl alcohol to benzaldehyde. Density functional theory calculations were used to rationalize the catalyst structure and elucidate the reaction mechanism, unveiling the role of the photogenerated holes in lowering the reaction energy barriers. Time-resolved transient spectroscopic studies were used to monitor the dynamics of photogenerated charges in the reaction, revealing the lifetimes and behaviors of excited states within the catalyst. Specifically, the introduction of silver atoms led to a significant enhancement in the excited state lifetime, which favors the hole-transfer in the presence of the benzyl alcohol. This indicated that the photoexcited carriers were effectively transferred to the reactant, thereby driving the oxidation process in the presence of oxygen. These mechanistic insights are pivotal in spectroscopically elucidating the reaction mechanism and can be practically applied to design single-atom photocatalysts more rationally, targeting materials that combine both rapid reductive quenching and efficient charge transfer to the metal.

## 1 Introduction

Understanding charge dynamics is essential for optimizing the efficiency and performance of photocatalysts, as these dynamics critically influence the separation and migration of photogenerated charge carriers.^1,2^ Advanced spectroscopic methods, particularly time-resolved transient spectroscopy, have become invaluable tools for probing charge carrier dynamics across various time scales, providing deep insights into the transient behaviors of photoexcited states.^1,2^ Despite these advancements, a comprehensive understanding of charge dynamics remains elusive for many photocatalytic processes. One such process where a detailed understanding could significantly enhance the catalytic performance is the selective oxidation of benzyl alcohol to benzaldehyde, a key reaction for producing intermediates used in the synthesis of fragrances, perfumes, and dyes.^3−5^ Traditionally, this transformation relies on stoichiometric oxidants, such as dichromate and permanganate, which require high temperatures and/or pressures, and generate significant amounts of toxic waste. To overcome these challenges, the adoption of molecular oxygen as a clean and cheap oxidant,^6^ the utilization of light to reduce dependence on nonrenewable resources,^7^ and the implementation of heterogeneous catalysts activated by light have been proposed.^8^ These approaches collectively minimize the need for toxic chemicals and energy-intensive conditions, offering a more sustainable solution for chemical processes.

Various photocatalysts based on TiO_2_ have been extensively studied.^9−11^ However, surface recombination of photoinduced charge carriers can slow reaction kinetics and reduce the product selectivity. Additionally, the limited photoactivation of TiO_2_-based photocatalysts outside the UV region restricts their versatility. Considerable efforts have been invested in photocatalysts that extend their photoresponse into the visible light spectrum.^4,12−15^ Adjusting the spectral window is critical because visible light comprises a substantial portion of the solar spectrum. In this direction, conjugated polymers have emerged as a good class of visible-light-driven semiconductors with a photoactive π-conjugation structure and high specific surface area. Carbon nitrides are a class of stable polymers that serve as an excellent support for these applications, offering a stable and efficient platform for harvesting visible light due to their unique electronic structure and ability to stabilize reactive sites.^16,17^ These materials facilitate efficient charge separation and migration, enhancing their photocatalytic activity. Additionally, the incorporation of metal atoms into carbon nitride matrices can further boost their photocatalytic performance by adjusting their bandgap and light absorption, which in turn optimize reaction kinetics.^18−21^ Such single-atom-decorated photocatalysts are gaining particular attention because they offer the advantage of maximizing the utilization of each metal atom while minimizing the amount of metal needed.^22−26^ To date, there has been no report of a high-performing single-atom catalyst for the photocatalytic oxidation of benzyl alcohol to benzaldehyde. Specifically, a copper single-atom catalyst was very recently reported for benzyl alcohol oxidation,^27^ but it achieved a turnover number (TON) below 100 and an isolated yield of only 24%. The proposed mechanism involved copper atoms absorbing light and transferring electrons to graphitic carbon nitride. However, the data showed no new spectral features, and the lack of observed changes in the spectrum raised questions about the efficiency of charge transfer. Therefore, further investigations, including time-resolved measurements, could provide additional insights into the underlying mechanisms and potential improvements.

This paper reports the first use of silver-based single-atom catalysts for the light-driven transformation of benzyl alcohol to benzaldehyde and aims to elucidate the mechanisms underlying the catalytic activity of these SACs by employing advanced spectroscopic techniques, such as transient absorption spectroscopy, alongside density functional theory (DFT) calculations. These findings are expected to contribute to the development of highly efficient and selective photocatalysts for visible light applications, advancing sustainable energy conversion and synthetic chemistry technologies.

## 2 Materials and Methods

### 2.1 Catalyst Synthesis

The Ag-based single-atom catalyst was prepared by reacting an aqueous solution of AgNO_3_ (1.70 g, 10 mmol; in 10 mL of deionized water) and an aqueous solution of sodium tricyanomethanide (1.13 g, 10 mmol; in 10 mL of deionized water) together, stirring the obtained solution for 3 h at room temperature. During this stage, a white precipitate of silver(I) tricyanomethanide was formed, which was filtered off, rinsed with water (3 × 10 mL), and dried in a vacuum (7 mbar, 50 °C). The obtained solids (37 mg, 0.015 mmol) were mixed with a 40% aqueous dispersion of 12 nm SiO_2_ particles (7.5 g, Ludox HS40) and cyanamide (3.0 g, 70 mmol), and the mixture was stirred at 70 °C for 16 h until complete water evaporation. The resultant white material was then ground and calcined at 550 °C for 4 h, using a heating ramp of 2.2 °C min^–1^. Silica template etching was performed by washing the obtained solids with an aqueous NH_4_HF_2_ solution (12 g in 50 mL of deionized water) at room temperature for 24 h. The resulting powders were filtered, washed three times with water and ethanol until a neutral pH was obtained, and finally dried under vacuum at 60 °C overnight. The reference CN_*x*_ sample was prepared following the same procedure, without the addition of silver salts.

### 2.2 Catalyst Characterization

Powder X-ray diffraction (XRD) was performed on a Philips model PW3040/60 X-ray diffractometer using Cu Kα radiation (λ = 0.15418 nm). Nitrogen physisorption measurements were performed after degassing the samples at 150 °C for 20 h using a Micromeritics 3Flex porosimeter at 77 K. The specific surface areas were calculated by applying the Brunauer–Emmett–Teller (BET) model to adsorption isotherms for 0.05 < *p*/*p*_0_ < 0.3 using the QuadraWin 5.05 software package. The pore size distribution was obtained by applying the quenched solid density functional theory model for N_2_ adsorbed on carbon with a cylindrical pore shape at 77 K. Elemental CHNS analysis was accomplished by combusting the samples using a Vario Micro device. The inductively coupled plasma optical emission spectroscopy (ICP–OES) studies were performed using a HORIBA Ultra 2 setup equipped with photomultiplier tube detection. X-ray photoelectron spectroscopy (XPS) was conducted using a Physical Electronics Instruments Quantum 2000 spectrometer with monochromatic Al Kα radiation generated from an electron beam operated at 15 kV and 32.3 W. All spectra were referenced to the C 1*s* peak of adventitious carbon at 284.8 eV. Ultraviolet photoelectron spectroscopy (UPS) spectra were acquired with a He I (21.2 eV) radiation source. The detector was a combined lens with an analyzer module thermoVG (TLAM). UV–vis absorption spectroscopy was carried out by collecting absorption spectra with a spectrophotometer (PerkinElmer LAMBDA 1050) and a continuous-flow static exchange gas cryostat (Oxford Instruments) equipped with three concentric chambers. High-angle annular dark-field scanning transmission electron microscopy (HAADF-STEM) imaging was carried out using an UltraSTEM100 microscope (Nion Co.) equipped with a cold field emission electron source and a quadrupole-octupole aberration corrector in the probe-forming electron optics, operated at 60 keV. The convergence semi-angle was set to 31 mrad, and the HAADF detector collection semi-angles were 90–190 mrad. The X-ray absorption spectroscopy (XAS) for the Ag *K* edge was measured at the Pohang Light Source-II (PLS-II) 8C Nanoprobe XAFS beamline (BL8C). A double-crystal monochromator with a Si(111) crystal was used to produce a monochromatic X-ray beam. The slits used in all measurements had an aperture of 0.5 mm (vertical) × 1 mm (horizontal). Gas ionization chambers were used as the detectors for the measurements in transmission mode. The XAS data analysis was done by using Demeter software (version 0.9.26), including background subtraction, normalization, plotting, and data fitting for extended X-ray absorption fine structure (EXAFS) spectra. Transient absorption spectroscopy (TAS) studies were collected in transmission geometry. An amplified femtosecond laser (Light Conversion Pharos) generated pulses of ca. 280 fs centered at 1030 nm. A probe broadband white light, spanning from 500 to 950 nm, was thus generated by focusing the pulses into a thin sapphire plate. At short delays (<5 ns), the third harmonic of the fundamental provided the pump light (343 nm) with a fluence of 1 × 10^19^ cm^–3^. At long delays (>1 ns), pump light at 355 nm, with a fluence of 1 × 10^19^ cm^–3^, was provided by the third harmonic of a Q-switched Nd YVO4 laser (Innolas Picolo), which was electronically triggered and synchronized to the femtosecond laser via an electronic delay. Photoluminescence (PL) measurements were conducted by using a 343 nm laser with a fluence of 1 × 10^18^ cm^–3^, and the signal was collected by a Maya 2000 pro spectrometer from Ocean Optics. Overall, all measurements were performed on a solution of 5 mg mL^–1^ of powder in acetonitrile in a 1 mm path quartz cuvette stirred before the measure. For the measurements in an anhydrous and anoxic environment, the powder was kept under vacuum for 2 h and subsequently heated at 130 °C for 2 h in nitrogen ambient before being dispersed in anhydrous acetonitrile.

### 2.3 Catalytic Tests

The photocatalytic oxidation of benzyl alcohols was performed using an Illumin8 parallel photoreactor (Asynt, UK), equipped with DrySyn OCTO MINI 8-position reaction station, standard hot plate magnetic stirring, and a cooling system to control reaction temperature. In a typical procedure, benzyl alcohol (19.5 mg, 0.18 mmol) was mixed with 5 mL of solvent and 5 mg of catalyst. The reaction flask was irradiated with blue light (λ = 450 nm, with 10 W LED COB chips) under continuous stirring (400 rpm) for the whole reaction time. All reactions were performed at 30 °C, varying the reaction time between 5 and 240 min. Control experiments with and without catalyst were performed in the dark under similar experimental conditions. Mechanistic studies were carried out with the addition of 1 equiv KI and CCl_4_ as hole and electron scavengers, respectively, with a reaction time of 30 min. For the continuous-flow experiments, catalyst (5 mg) and 50 μm glass beads (3 g) were mixed in a vortex generator and packed in a fluorinated ethylene-propylene (FEP) tube (500 mm long, 3.2 mm o.d., and 2.1 mm i.d.). The reactor was then plugged with a quartz wool filter at both ends to prevent the catalyst from leaking and connected via 1/8″ o.d. 1/4″ 28 flat bottom flangeless fittings to the other 1/16″ o.d. tubing. The reactor volume was calculated as dead volume using the difference between the dry packed-reactor mass and the mass of the packed reactor filled with the reaction solvent. A solution of benzyl alcohol (37 mmol L^–1^ in acetonitrile, MeCN), was introduced by a syringe pump (Harvard PHD ULTRA CP) operating at quasi-ambient pressure into the assembled packed-bed photoreactor. Reaction products were analyzed via gas chromatography (GC) equipped with a flame ionization-mass spectrometer (MS) detector. The detection was done using two columns, HP-INNOWAX (5 m × 0.250 mm × 0.15 μm) and DB-5MS (20 m × 0.180 mm × 0.18 μm) connected in series. Quantification was performed after the calibration of the GC–MS with commercial standards.

### 2.4 Computational Details

Spin-polarized DFT calculations were performed with the VASP code,^28−30^ using the generalized gradient approximation, as implemented in the Perdew–Burke–Ernzerhof (PBE) functional.^31^ Dispersion forces have been included according to the Grimme’s D3 parametrization.^32^ The valence electrons have been expanded on a set of plane waves with a kinetic energy cutoff of 400 eV, whereas the core electrons were treated with the projector augmented wave approach (PAW).^33,34^ The threshold criteria for electronic and ionic loops were set to 1 × 10^–5^ eV and 1 × 10^–2^ eV Å^–1^, respectively. The sampling of the reciprocal space was reduced to the gamma point because of the cell size. Single-point PBE0^35,36^ calculations were performed on top of PBE-optimized structures to improve the description of the electronic structure. This strategy allows us to avoid intensive geometry optimizations with hybrid functionals with an acceptable error bar of about 0.1 eV.^37^ We considered a corrugated CN_*x*_ layer characterized by heptazine pores and a newly elucidated structure of carbon nitride, which some of us recently validated through experimental results and DFT simulations.^38^ The optimized lattice parameters for the CN_*x*_ with heptazine pore are *a* = 13.767 Å, *b* = 11.445 Å, γ = 122°, while the lattice parameters for the new structure of CN_*x*_ are *a* = 15.593 Å, *b* = 20.698 Å, γ = 90.2°. The binding energies (Δ*E*) of each intermediate were calculated with respect to the free molecular species and catalyst. The Gibbs energies (Δ*G*) were evaluated by adopting the thermochemistry approach of Nørskov and co-workers including zero-point energy correction and entropy terms.^39−41^ The first were calculated within the harmonic approximation. Entropies of gases were taken from the international tables, and the entropy of solid-state species was considered equal to zero.^41^

## 3 Results and Discussion

### 3.1 Catalyst Characterization and Proof of Catalytic Performance

**Figure 1:**
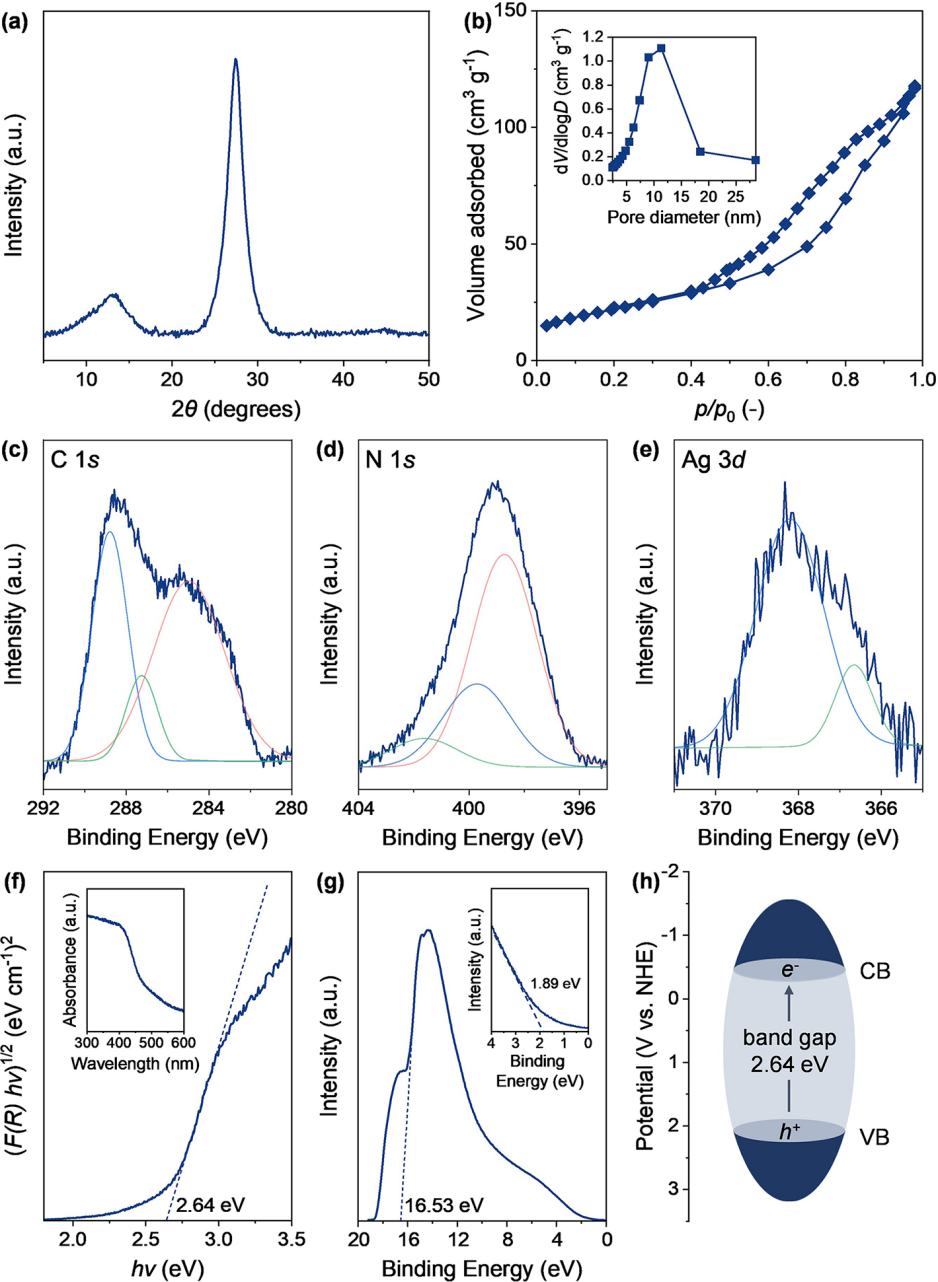
(a) X-ray diffraction pattern, (b) N_2_ physisorption isotherm with the corresponding pore size distribution calculated using the BJH method from the desorption branch as an inset, (c) C 1*s*, (d) N 1*s*, and (e) Ag 3*d* X-ray photoelectron spectroscopy of Ag_1_@CN_*x*_. (f) Tauc plot of Ag_1_@CN_*x*_, with the relative UV–vis absorption spectra as an inset. (g) UPS spectra of Ag_1_@CN_*x*_, with the extended UPS spectra of the materials as an inset. (h) Band structure of the material, obtained from UPS and UV–vis absorption spectroscopy.

We commenced the study by characterizing the Ag-based single-atom catalyst (Ag_1_@CN_*x*_) to determine its properties and prove that the Ag species were atomically dispersed. The crystallinity and purity of the prepared catalyst were evaluated through XRD analysis (Figure 1a). In the diffractogram, two main diffraction peaks at 2θ = 13 and 28° were found, corresponding to the *N*-linkage of the tri-*s*-triazine motif and π–π stacking of aromatic structures in the support, respectively.^42^ Porosity, pore diameter, and surface area of the material were determined by N_2_ physisorption (Figure 1b and Table S1). In particular, the analysis evidenced the relatively high BET surface area of Ag_1_@CN_*x*_ (174 m^2^ g^–1^), consistent with previous reports,^43^ and the mesoporous nature of the fabricated material. The elemental composition of the catalyst was elucidated by CHNS and ICP–OES studies (Table S1). The C/N ratio, resulting from the CHNS analysis, was close to 0.65, which is the reference value for tri-*s*-triazine-derived CN_*x*_ carriers.^44^ The ICP–OES analysis proved the effective incorporation of the metal on the support, with a metal loading of 0.3 wt %.

XPS analysis was employed to evaluate the oxidation state of the elements (C, N, and Ag) constituting our catalyst (Figure 1c–e). The XPS C 1*s* spectrum presented three peaks: the first (285.1 eV, in red) was assigned to both adventitious carbons and energy calibration *sp*^2^ carbon; the second (287.3 eV, in green) was related to *sp*^3^—C≡N bonds;^43^ the last one (288.8 eV, in cyan) corresponded to N–C=N carbons in the aromatic structure of the carbon nitride.^45^ In the N 1*s* spectrum, three peaks were derived from the deconvolution of the experimental signal. The peak at 398.8 eV (in red) was related to the C=N–C nitrogen in the pyridinic structure of CN_*x*_; the one at 399.8 eV (in cyan) was assigned to the presence of tertiary C=N–C_3_ nitrogen. The peak at 401.6 eV (in green) corresponded instead to primary amine groups coming from polymerization defects in the carrier. The XPS Ag 3*d* spectrum consisted of two contributions, the main at 368.2 eV (in cyan) and a minor one at 366.7 eV (in green), characteristic of Ag^+^ and Ag^0^, respectively. The presence of Ag^0^ may result from the partial reduction of the sample under the ultrahigh vacuum conditions of XPS. For comparative purposes, XPS analysis was also performed on the bare CN_*x*_ sample (Figure S1). While minor variations were observed in the N 1*s* spectra when comparing Ag_1_@CN_*x*_ to bare CN_*x*_, it is challenging to definitively attribute these changes to N–metal binding due to the complexity of the nitrogen environment in carbon nitride. Indeed, the multiple nitrogen species, such as pyridinic, pyrrolic, and graphitic nitrogen, may exhibit subtle variations upon interaction with metal atoms. However, the observed shifts in the N 1*s* spectra were relatively minor and could also be influenced by factors such as changes in the electronic structure of CN_*x*_ caused by the embedding of Ag atoms, or by alterations in the local bonding environment unrelated to direct N–metal coordination. To determine the photophysical properties of Ag_1_@CN_*x*_ and identify the band structure, UV–vis measurements were performed (inset of Figure 1f) and showed the wide absorption band of the single-atom catalyst at wavelengths greater than 400 nm. The band gap energy was estimated at 2.64 eV from the Tauc plot obtained from the UV–vis spectra (Figure 1f). Additionally, UPS spectroscopy was employed to elucidate the exact valence band of Ag_1_@CN_*x*_ (Figure 1g), which was determined to be at +2.14 eV. Considering the calculated band gap energy, the conduction band energy was estimated at −0.5 eV (Figure 1h).

**Figure 2:**
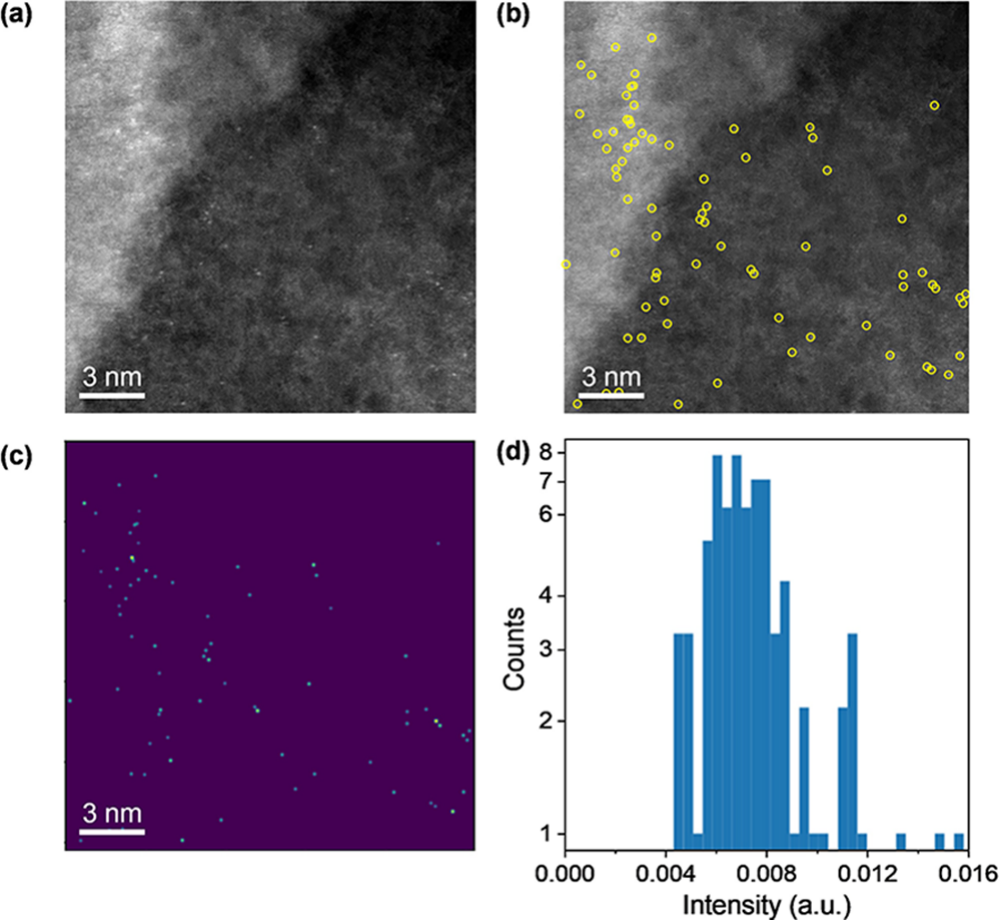
(a) Atomically resolved HAADF-STEM micrograph of Ag_1_@CN_*x*_ with (b) single atoms identified by peak finding labeled in yellow. (c) Fitting of a two-dimensional Gaussian and the background intensity at each atom position marked in (b) retrieves the intensity of the single-atom features. (d) Histogram of relative intensities for Ag_1_@CN_*x*_.

Finally, using high-resolution HAADF-STEM, we could distinctly observe individual metal atoms uniformly dispersed on the support (Figure 2). The micrographs revealed isolated bright spots corresponding to single silver atoms, confirming their dispersion at the atomic level. Furthermore, analysis of the STEM images indicated a nearly homogeneous distribution of single silver atoms across the support surface, proving that that the catalyst synthesis effectively prevented the agglomeration of silver atoms into larger clusters and nanoparticles.

**Figure 3:**
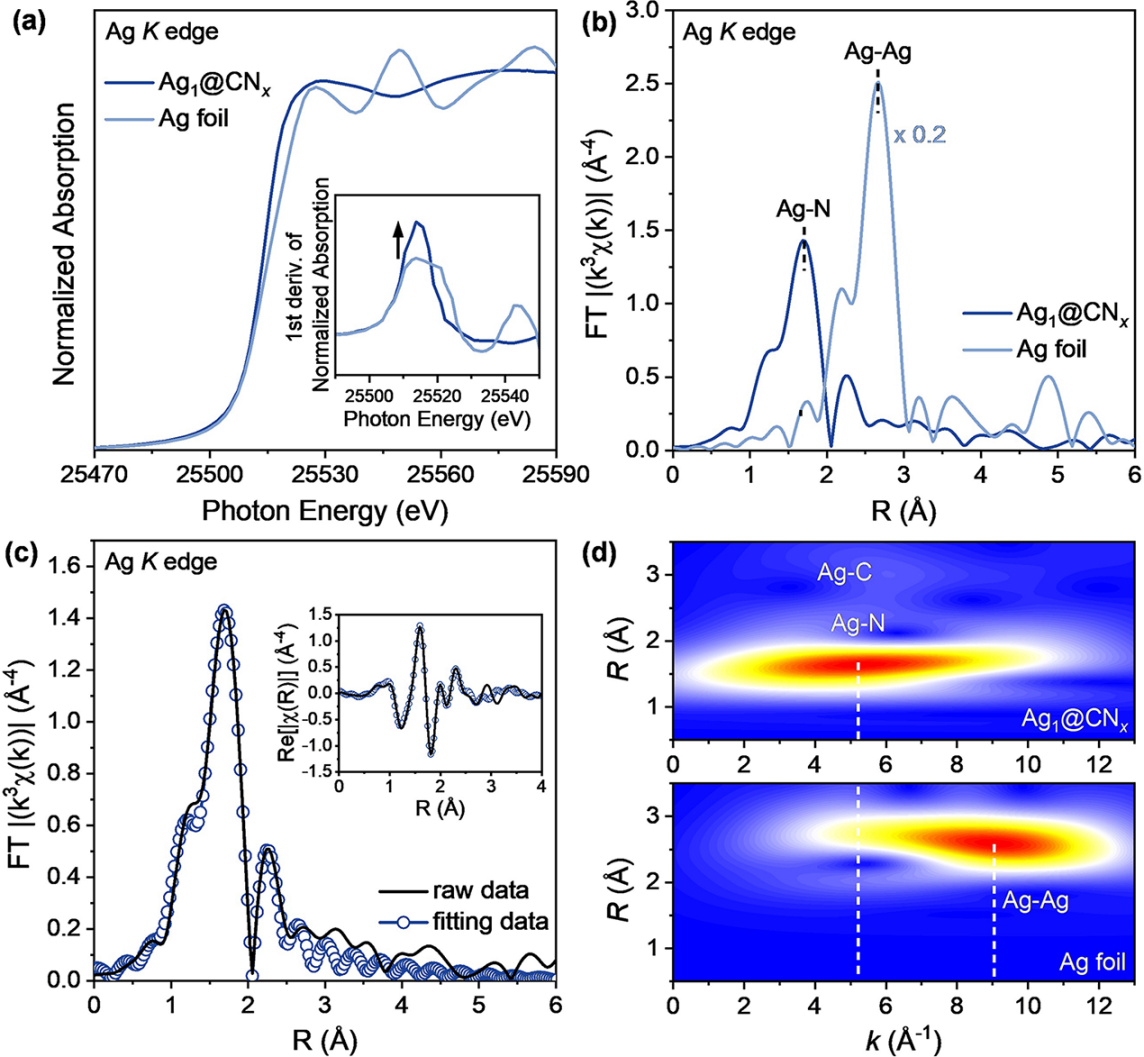
X-ray absorption spectroscopy data of Ag *K* edge for Ag_1_@CN_*x*_ and Ag foil. (a) Ag *K* edge XANES spectra, (b) non-phase corrected *k*^3^-weighted FT-EXAFS spectra, (c) EXAFS fitting data for Ag_1_@CN_*x*_, and (d) corresponding WT-EXAFS data.

The electronic states and local structure of Ag_1_@CN_*x*_ were probed using X-ray absorption spectroscopy. Figure 3a shows the Ag *K* edge of the X-ray absorption near edge structure (XANES) spectra of Ag_1_@CN_*x*_ and Ag foil, revealing the valence states around the Ag atoms. The distinct XANES shapes of Ag_1_@CN_*x*_ compared with Ag foil were due to charge transfer from the Ag atom to the carbon nitride framework. The first derivative of the XANES for Ag_1_@CN_*x*_ and Ag foil is displayed in the inset, with the increased edge intensity observed for Ag_1_@CN_*x*_. The non-phase corrected *k*^3^-weighted Fourier-transformed extended X-ray absorption fine structure (FT-EXAFS) is shown in Figure 3b.

An apparent peak at 1.6 Å was observable in the non-phase corrected Fourier-transform EXAFS data of Ag_1_@CN_*x*_, contrasting with Ag foil, which showed the presence of Ag–Ag bonds with a distance of approximately 2.6 Å. A fitting process, which involves analyzing quantitative values such as bond distance and coordination number, was also performed (Figure 3c). The fitting model derived by density functional theory (vide infra) agreed well with the raw data, indicating that the theoretical model for Ag_1_@CN_*x*_ closely matched the obtained EXAFS data. The summarized EXAFS fitting results are in Table S2. In brief, the main peak of Ag_1_@CN_*x*_ at 1.6 Å in the non-phase corrected Fourier-transform EXAFS corresponded to two Ag–N paths: one at 2.02 Å for short Ag–N and another at 2.2 Å for long Ag–N. This fitting result indicates that Ag atoms were surrounded by four atoms with a distorted coordination, rather than a planar-type Ag–N_4_ structure, and the longer Ag–N distance was similar to previously published values.^46^ Moreover, the minor contribution near 2.2 Å in the non-phase corrected Fourier-transform EXAFS matched with Ag–C with a bond length of 2.5 Å. The presence of the Ag–C path suggests a possible longer-range interaction between Ag and C atoms, attributed to the local structure of the carbon nitride matrix. However, the presence of this longer-range Ag–C path does not necessarily confirm the existence of direct chemical bonds and is not expected to play a primary role in the bonding or catalytic activity of the Ag sites. As a result, the EXAFS fitting for Ag_1_@CN_*x*_ showed evidence that the catalyst had a single-atom structure with nitrogen atoms without Ag–Ag formation. In turn, wavelet-transformed (WT)-EXAFS analysis was employed to resolve the *k*-space and *R*-space of the FT-EXAFS data to gain a better understanding of the bonding (Figure 3d). In the WT-EXAFS data, the position of maxima in *k*-space depends on the scattering atoms bonded to the central Ag atoms. Bonding with heavier or lighter atoms shows maximum points at higher or lower *k*-space regions in the WT-EXAFS data. For example, Ag foil with Ag–Ag bonding shows a maximum point near 9 Å^–1^, while Ag_1_@CN_*x*_ shows a maximum point near 5.2 Å^–1^, with no other maxima at higher *k* regions. This analysis revealed that the surroundings of Ag_1_@CN_*x*_ had a bonding structure with only light elements, such as N and C, rather than Ag. Therefore, the WT-EXAFS data further supported the conclusion that Ag_1_@CN_*x*_ contains only isolated silver atoms.

The Ag_1_@CN_*x*_ catalyst was exploited in the photocatalytic conversion of benzyl alcohol to benzaldehyde. Initial control experiments were conducted without light and without photocatalyst (Table 1, entries 1 and 2), showing no reaction and confirming that both components are essential for the catalysis. To understand the role of molecular oxygen in the catalytic cycle, we performed the photo-oxidation after degassing the reaction vial, thus under anoxic conditions (Table 1, entry 3). The catalytic results showed a significantly reduced turnover number (TON), equal to 168 mmol_prod_ mmol_Ag_^–1^, proving that the presence of oxygen was crucial for the formation of the product. When optimal conditions were restored, including blue light irradiation, Ag_1_@CN_*x*_, and an air atmosphere, the reaction achieved a TON of 687 mmol_prod_ mmol_Ag_^–1^. These results confirmed that the synergistic effect of light, photocatalyst, and molecular oxygen was essential for achieving high catalytic turnover numbers in the photo-oxidation process.

**Table 1:**
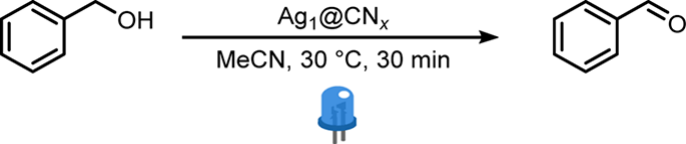
Control Experiments for the Light-Driven Benzyl Alcohol Oxidation

entry	control experiment^a^	TON (mmol_prod_ mmol_Ag_^–1^)^b^
1	no light	0
2	no photocatalyst	0
3	after N_2_ degassing of the reaction vial	168
4	with no variations from the standard conditions	687

a Unless specified otherwise, the reaction was conducted at a benzyl alcohol concentration of 37 mmol L^–1^, in MeCN, at 30 °C, for 30 min, using Ag_1_@CN_*x*_ as photocatalyst (5 mg), and blue (450 nm) light.

b The quantity of benzaldehyde mmol was determined via GC–MS using a calibration curve.

**Figure 4:**
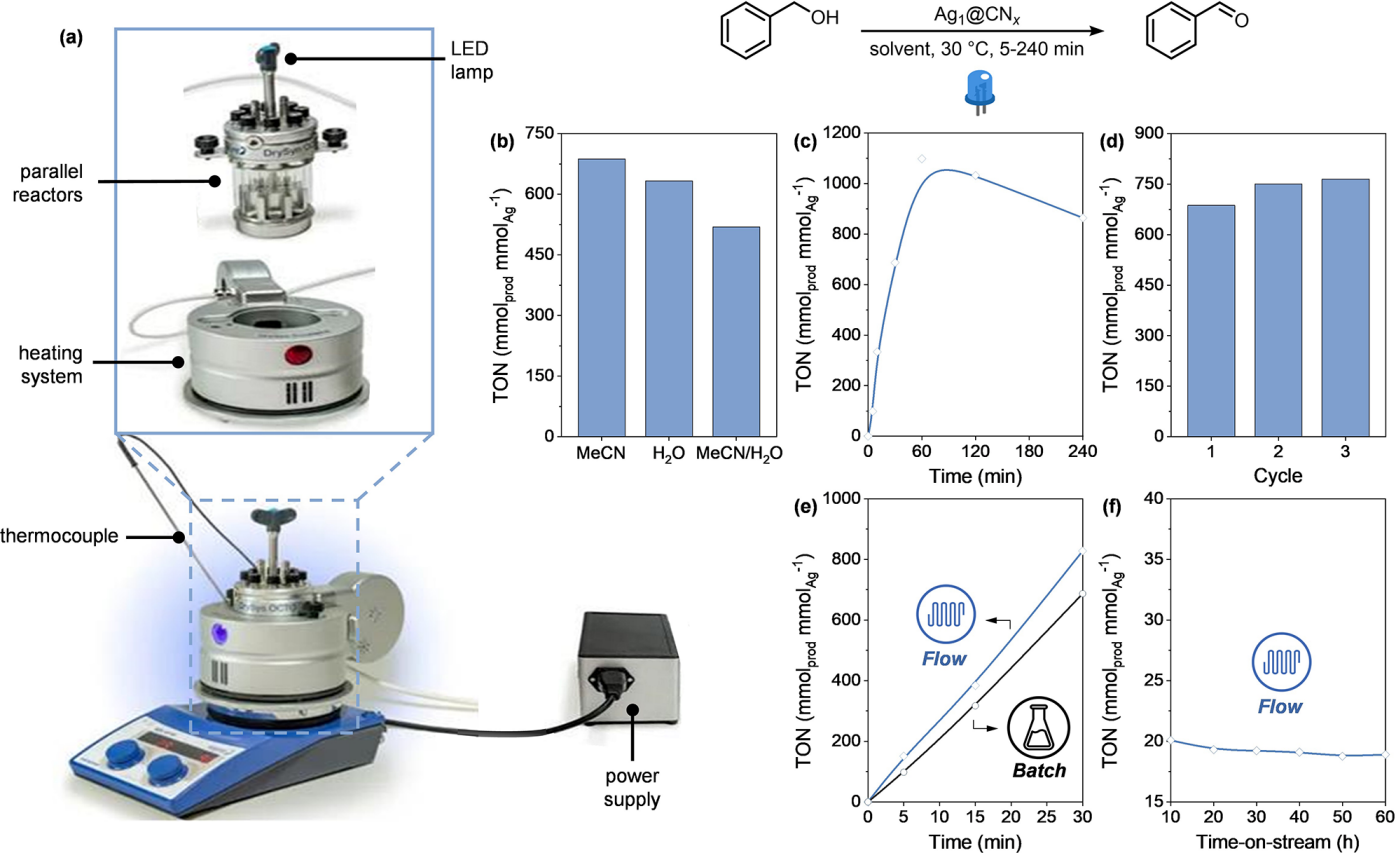
(a) DrySyn Octo-Mini reaction station for the parallel batch screening of catalysts and catalytic conditions. (b) Effect of the solvent type, (c) time-dependent rate, and (d) recycling experiment for the light-driven benzyl alcohol oxidation. (e) Comparison between batch and flow oxidation of benzyl alcohol and (f) continuous-flow time-on-stream experiments. Unless specified otherwise, the batch experiments were conducted at a benzyl alcohol concentration of 37 mmol L^–1^, in various solvents, at 30 °C, using Ag_1_@CN_*x*_ as photocatalyst (5 mg), and blue (450 nm) light. The flow experiments were conducted at a benzyl alcohol concentration of 37 mmol L^–1^, in MeCN, at 30 °C, using Ag_1_@CN_*x*_ as photocatalyst (5 mg), glass beads (3 g) as inert material, and blue (450 nm) light. Results were determined via GC–MS using a calibration curve.

To probe the effect of the solvent properties on the reaction progress, several solvents were then examined using visible-light irradiation (450 nm) for 30 min, using the parallel photoreactor system in Figure 4a. Specifically, the solvents chosen for this study included acetonitrile, cyrene, and water, due to their reputation as more environmentally friendly alternatives compared to traditionally used organic solvents.^47^ This selection aligns with the growing interest in green chemistry, which aims to reduce the environmental impact of chemical processes by using safer solvents. The catalytic results (Figure 4b) showed that the TONs obtained with acetonitrile and cyrene were relatively close, at 687 and 633 mmol_prod_ mmol_Ag_^–1^, respectively. This similarity can be explained by the comparable polarities of the two solvents, with acetonitrile having a relative polarity of 0.46 and cyrene of 0.33,^48^ suggesting that both solvents provide a similar environment for the reaction. On the other hand, the use of water as solvent resulted in a slightly lower TON of 519 mmol_prod_ mmol_Ag_^–1^, which can be justified by the moderate solubility of the product in water.^49^ Overall, polarity appeared as a critical factor in solvent selection because it influenced the solubility of reactants and products, as well as the reaction kinetics and mechanism. We also studied the effect of the reaction time on the product formation. For this purpose, we utilized the same batch reactor, which enabled continuous monitoring of product evolution without interrupting the irradiation of the reaction vessel. Upon increasing the reaction time to 60 min, benzyl alcohol showed complete conversion, and the benzaldehyde TON reached 1098 mmol_prod_ mmol_Ag_^–1^ (Figure 4c), showing a 10-fold increase in comparison to reported protocols for the selective photo-oxidation of benzyl alcohol (Table S3). The steady increase in product formation over time indicates a consistent catalytic performance, suggesting that the Ag single atoms maintain their stability under the reaction conditions. However, beyond 60 min, we observed the overoxidation to benzoic acid, which reduced the selectivity for benzaldehyde, and therefore the TON. To further evaluate the stability of the Ag-based catalyst, we conducted a recyclability test over three reaction cycles. After each 30 min oxidative reaction, the catalyst was filtered, washed, dried, and reused. As depicted in Figure 4d, there was no significant deviation in the TONs across multiple cycles, indicating that the catalyst maintained its chemical and structural integrity during the photo-oxidation process. We further validated the catalyst stability through a detailed characterization of the used catalyst which exhibited a similar density of single Ag atoms and no changes in the catalyst’s structure, as shown in the Supporting Information (Figure S2). To benchmark the performance of the Ag_1_@CN_*x*_ photocatalyst and illustrate the advantages of using a fully integrated Ag-doped single-atom system, we then investigated the activity of metal-free CN_*x*_ under the optimal reaction conditions previously identified (Table S4). The absence of Ag resulted in a significantly reduced photocatalytic performance, highlighting its essential role in the catalysis. The superior performance of the single Ag atom-doped material has been attributed to ligand-to-metal charge transfer (LMCT) phenomena. These phenomena are supposed to enhance electron transfer from the support to the metal surface, thereby increasing the electron flux from the catalyst to the substrate. This enhanced electron transfer is critical for improving catalytic efficiency and achieving higher yields. Absorption spectra of the CN_*x*_ and Ag_1_@CN_*x*_ samples (Figure S3) do not exhibit significant differences at longer wavelengths, where an LMCT transition would typically be observed. This indicates that such a transition is not present in the ground state. However, a slight shift is noted in the emission spectra, which aligns with the formation of a charge transfer state in the excited state. With the optimal conditions established, we demonstrated the protocol flexibility by oxidizing various primary and secondary alcohols (Figure S4 and NMR data in the Supporting Information). We also focused on scaling up the process using continuous-flow technology and ensuring uniform radiation distribution inside the reactor. Compared to the batch photoreactor, the packed-bed reactor design and the porous framework of the mesoporous photocatalyst created an ideal environment to minimize mass transfer limitations in the multicomponent reaction. In particular, the use of glass beads as a copacking material in the packed-bed reactor facilitated catalyst particle separation and dilution, reducing the competition for visible-light photon absorption. Kinetic studies under these flow conditions showed an increased production of the desired model compound compared to the batch method (Figure 4e). This improvement, along with the catalyst stability (verified by time-on-stream analysis, Figure 4f) and the easy recyclability of the photocatalytic system, demonstrated the importance of continuous-flow reactor design for scaling up the SACs-based photocatalyzed process.

### 3.2 Mechanistic Insights, Operando Studies, and Trapping Experiments

Following the catalytic experiments, TAS, in the fs to μs time range, was employed to gain deeper insights into the mechanistic aspects of the photocatalytic process. TAS provides time-resolved information about the excited-state and photocarrier dynamics, enabling the identification of key electronic pathways involved in the chemical reaction.

**Figure 5:**
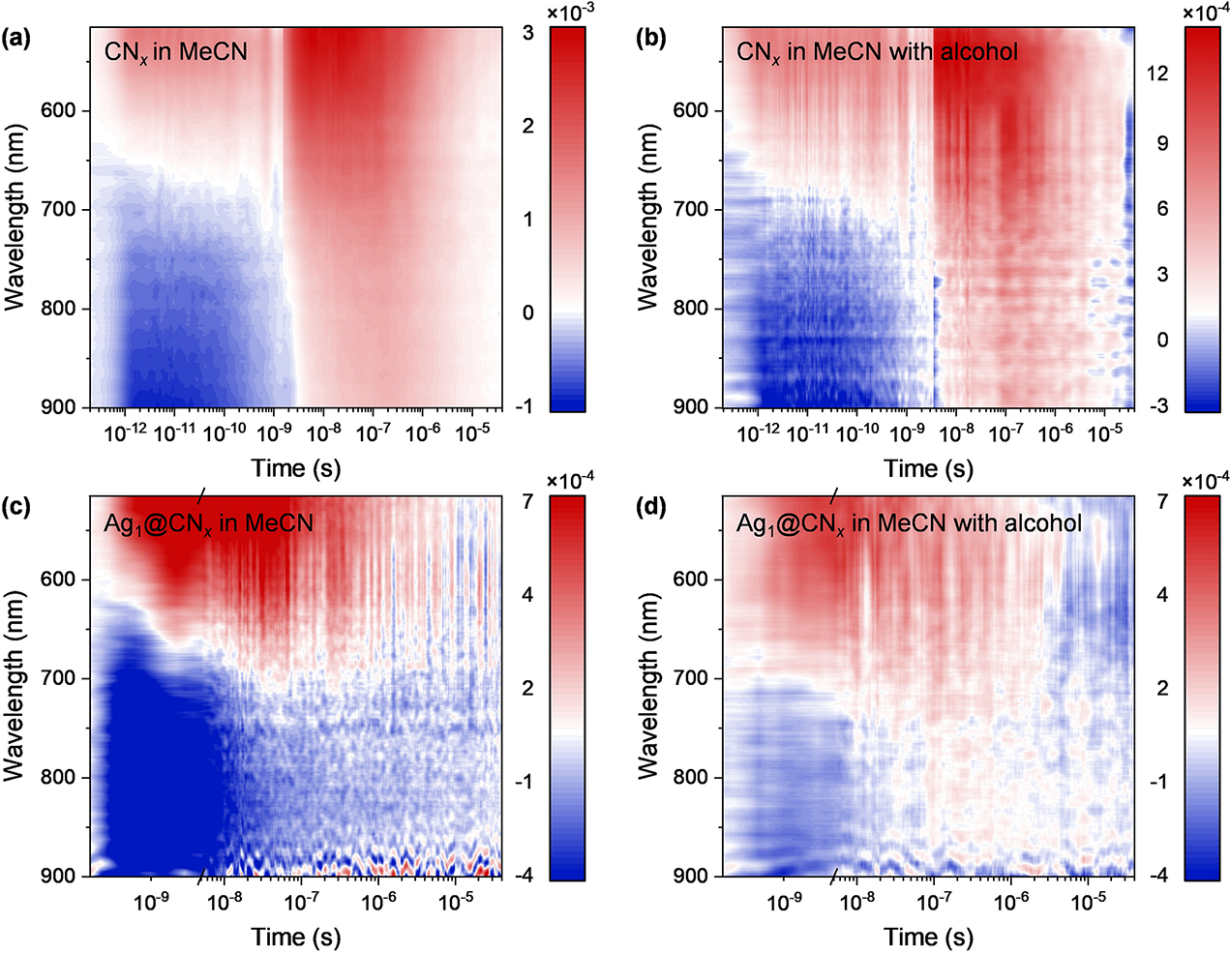
2D representation of the transient absorption spectra up to μs time scale on CN_*x*_ (a,b) and Ag_1_@CN_*x*_ (c,d) samples in acetonitrile without (a,c) and with (b,d) the addition of benzyl alcohol (37 mmol L^–1^). Areas shaded blue represent negative photoinduced absorption feature, red positive areas correspond to photo bleach signal.

The transmission spectrum of a white light probe pulse reveals the ground state absorption of the sample (pump-off). Then, the transmission is probed again when the pump pulse, energetic enough to deplete the ground state of the photoactive material, is absorbed by the sample (pump-on). The difference between the pump-off and pump-on transmission spectra (Δ*T*) allows to observe the presence of new photoinduced electronic transitions defined as photoinduced absorption bands (negative sign), PIA, or their suppression, defined as photobleach, PB (positive sign). By monitoring the Δ*T*/*T* spectral evolution at different time delays τ, we can then reconstruct the photocarriers dynamics from their generation until their recombination to the ground state. Figure 5 shows the heat maps collected from CN_*x*_ (Figure 5a) and Ag_1_@CN_*x*_ (Figure 5c) in the fs and μs time range, and from the same samples with the addition of alcohol (Figure 5b,d, respectively). In the map, the intensity of the absorption signal is depicted through a color gradient, ranging from blue, for negative signals (PIA bands), to red, for positive signals (PB bands). Thus, these maps allow for easy visualization of how absorption characteristics develop across the measured spectral and time intervals.

**Figure 6:**
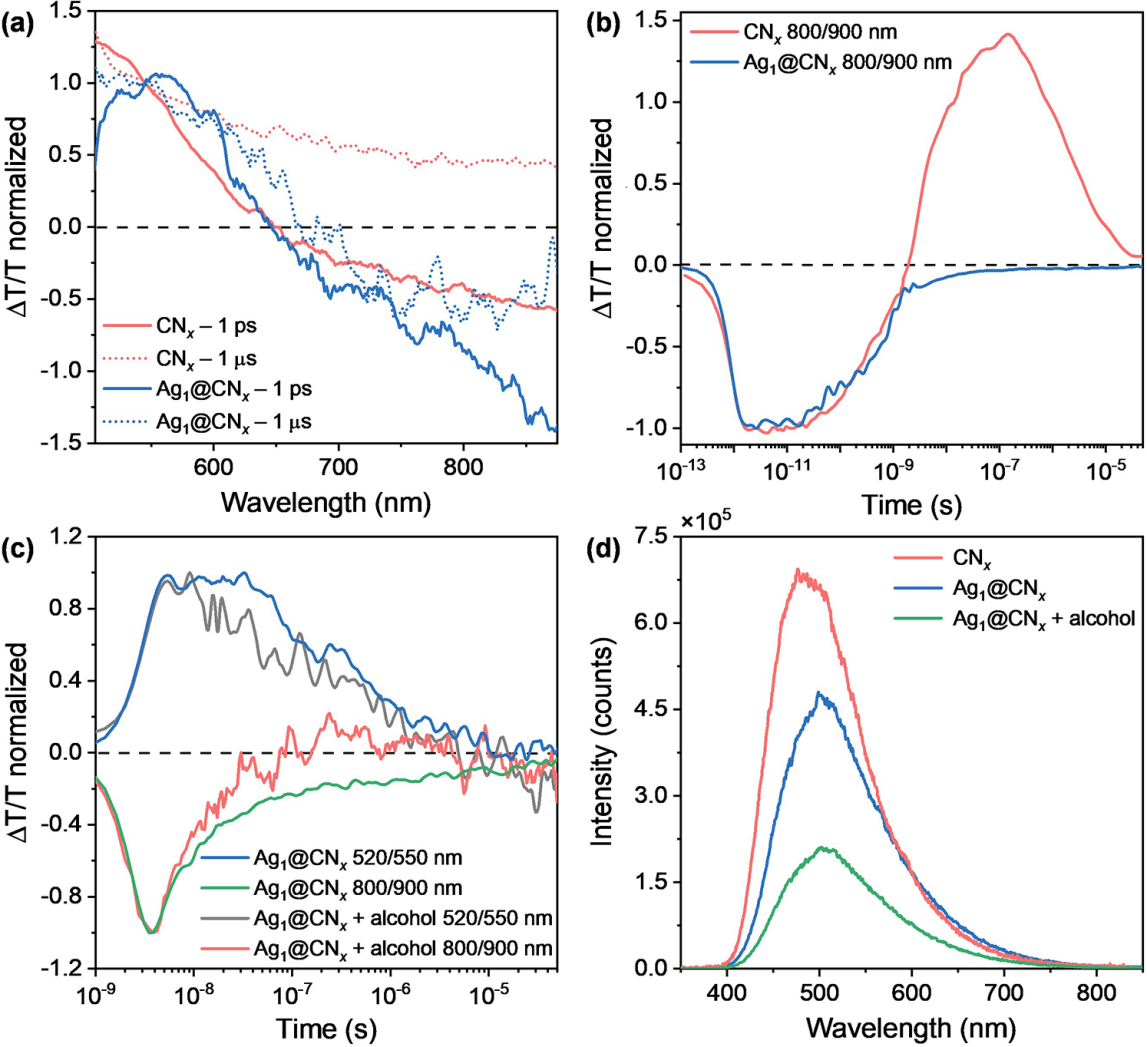
(a) Transient absorption spectra at 1 ps (solid line) and 1 μs (dashed line) after photoexcitation of CN_*x*_ (red line) and Ag_1_@CN_*x*_ (blue line). (b) Dynamics probed at 800–900 nm from CN_*x*_ (red line) and Ag_1_@CN_*x*_ (blue line), and (c) dynamics collected from μs-TA in the 520/550 and 800/900 nm spectral range on Ag_1_@CN_*x*_ in acetonitrile, with and without the addition of benzyl alcohol (37 mmol L^–1^). As evidenced, the sample with benzyl alcohol shows a faster decay of both the PIA (green and red line) and PB (black and blue) signals compared to the counterpart without substrate, suggesting an efficient carrier extraction from the scaffold to the reactant. (d) Comparison of static photoluminescence spectra of CN_*x*_ and Ag_1_@CN_*x*_, with and without the addition of benzyl alcohol (37 mmol L^–1^). The intensity of the signal is quenched with the introduction of benzyl alcohol due to the extraction of a part of the photoexcited carriers.

The presence of single Ag atoms does not affect significantly the light absorption spectrum of the light absorbing substrate (Figure S3). In fact, we measure two identical spectra corresponding to the one of CN_*x*_. This indicates that the presence of Ag does not induce the formation of a charge transfer state in the ground state. Figure 6a shows that both CN_*x*_ and Ag_1_@CN_*x*_ present a photobleach between 500 and 640 nm, in agreement with the optical bandgap of CN_*x*_, and a photoinduced absorption band PIA at longer wavelengths associated to the population of a photoexcited state in the CN_*x*_,^49^ (solid lines). The presence of Ag primarily affects the photoexcitation lifetime; in fact, only Ag_1_@CN_*x*_ shows a PIA band which persists in the microsecond range (Figure 6a, dashed lines), with its dynamic going to zero within tens of microseconds (Figure 6b). This indicates a charge transfer from the CN_*x*_ to Ag, sustained for μs.^50^ On the contrary, in the absence of Ag, the photoexcited states either recombine or get trapped in the defective substrate. In fact, in the CN_*x*_ sample we observe the PIA decay and the formation of a positive signal in the ns time window at wavelengths longer than those of the CN_*x*_ optical bandgap. Such a positive band is indicative of trapping sites within the CN_*x*_ bandgap.

When the dynamics are measured in an anhydrous and anoxic environment, no evident changes can be seen in the photocarrier lifetimes (Figure S6), suggesting that the charge transfer process between CN_*x*_ and Ag is not significantly influenced by ambient moisture or oxygen. This invariance indicates that the efficient charge separation observed at the silver interface is primarily dictated by the intrinsic electronic interactions between the materials, rather than being affected by surface passivation or competing reactions with adsorbed species.

The changes occurring in the Ag_1_@CN_*x*_ catalyst upon photoexcitation can be appreciated by the analysis of the electronic structure of the complex as obtained from DFT calculations. In the ground state the Ag atom is in a +1 oxidation state, as shown by the absence of spin density on the Ag center, a Bader charge of +0.7 e, and by the fact that in the DOS curves the 5s orbital is empty, at high energy. The photoexcitation process has been simulated by forcing the system to have a different spin multiplicity, an approximation typically adopted with DFT of solid-state systems to approximate excited states.^52,53^ From the eigenvalues, the bandgap excitation of Ag_1_@CN_*x*_ is 2.55 eV. The process results in the creation of a hole delocalized over the CN_*x*_ support, with no contribution from the Ag atom, and an excited electron which is also largely delocalized over the support but with partial contribution from the Ag orbitals (see the spin density plot and the DOS curves in Figure S7).

Finally, we performed the TA study in presence and absence of benzyl alcohol. Figure 6c shows a faster recovery of the Ag_1_@CN_*x*_ PIA in the presence of alcohol, while Ag-free CN_*x*_ photoinduced dynamics are not perturbed (Figure S8a). This can be interpreted as an efficient charge transfer from the scaffold to the alcohol in the ns time scale, also corroborated by the great reduction of PL intensity upon benzyl alcohol introduction, as shown in Figure 6d.

To summarize, the study of CN_*x*_ and Ag_1_@CN_*x*_ solutions show that a long living charge transfer state is formed between the organic and metallic moiety, which we spectrally identify in the PIA band of the TAS spectra. Then, when the alcohol is added, it effectively takes away the carrier from Ag_1_@CN_*x*_, resulting in a faster decay of the aforementioned PIA band (see also fitting results in Figure S8b). Overall, benzyl alcohol is shown to be able to efficiently extract carriers during its adsorption, making the dynamics faster and initiating the catalytic mechanism.

It must be emphasized that when molecular oxygen is involved, the number of possible reaction pathways in which charge carriers can help the reaction is very large. The ground state of the Ag_1_@CN_*x*_ is made by an Ag^+^ species with one electron donated the conduction band of the support. We simulated the photoexcitation of the SAC by computing a doublet-quartet excitation adopting the restricted spin approximation.^51,52^ The photoexcitation generates one hole in the valence band and one electron in the conduction band with energy cost of 2.5 eV, which explains the need for visible light excitation. The catalyst is not able to transfer the conduction band electron to an adsorbed O_2_ molecule with consequent formation of O_2_^–^, indicating that this process cannot occur and that holes could be the key species in lowering the barrier of the reaction. This is in line with trapping experiments, where the reaction was performed in the presence of a series of free radical scavengers (Table S5). In fact, the addition of KI (Table S5, entry 2), a scavenger for photogenerated holes (H^+^), completely suppressed the reaction, highlighting that photogenerated holes are essential for the selective oxidation of benzyl alcohol. This observation aligns with previous reports suggesting that holes activate benzyl alcohol, generating carbon-centered radicals.^27^ On the contrary, the addition of CCl_4_ (Table S5, entry 3) as an electron scavenger did not alter the catalytic results. Electron scavenging is expected to impede the formation of superoxide radicals (^•^O_2_^–^), as the reduction of O_2_ to ^•^O_2_^–^ relies on electron transfer, and thus, the unchanged catalytic performance indicates the absence of ^•^O_2_^–^ in the reaction mechanism.

**Figure 7:**
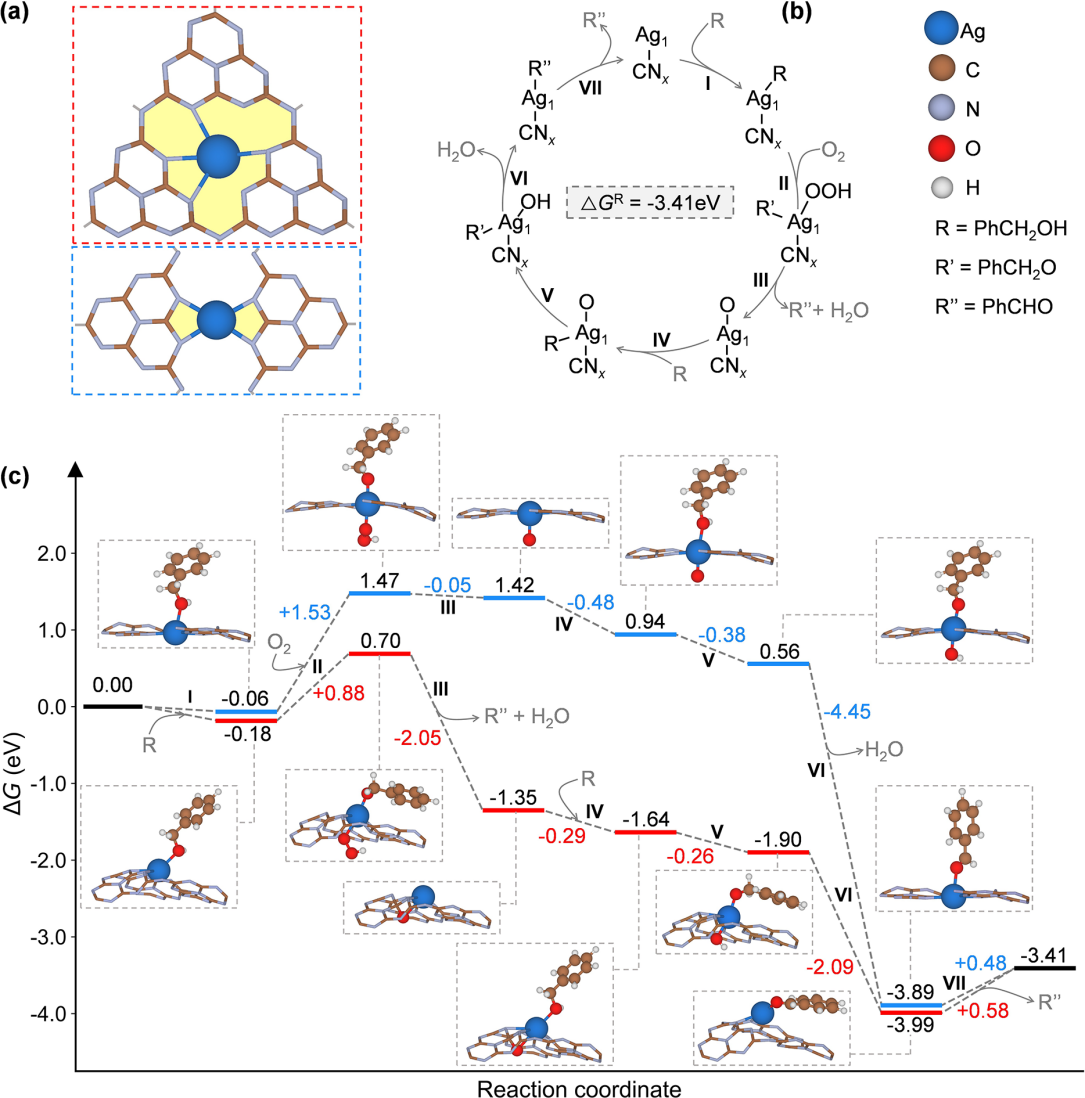
(a) Atomistic structure of Ag atom in the heptazine pore of CN_*x*_ (top, red dotted line) and the pore of the new structure of CN_*x*_ (bottom, blue dotted line), (b) mechanism of the oxidation of benzyl alcohol to benzaldehyde, and (c) Gibbs free energy profile for the oxidation of benzyl alcohol to benzaldehyde. Brown, blue, light purple, red, and white balls correspond to carbon, silver, nitrogen, oxygen, and hydrogen atoms. In the figures the roman numbers indicate the elementary steps of the process.

Based on the experimental insights, we modeled the oxidation of benzyl alcohol to benzaldehyde on the Ag single metal atom anchored to both CN_*x*_ catalyst; heptazine pore and triazine adduct (new structure). Experimental evidence highlighted the crucial role of oxygen for the formation of product (Table 1, entry 3), however to thoroughly investigate the possible reaction pathways, we followed two distinct reaction profiles: one explicitly considers the presence of molecular oxygen, where the overall reaction consumes one O_2_ molecule and two benzyl alcohol molecules releasing two benzaldehyde and two water molecules, while the second proceeds without it (Figure S9 and details in the Supporting Information). In both cases, the mechanistic cycle hypothesized that Ag_1_@CN_*x*_ photocatalyst was first excited by light. This photoexcited photocatalyst can undergo oxidative single electron transfer to enable the adsorption of benzyl alcohol and the oxidation of alcohol to aldehyde (Figure 7b). This reaction path agreed with previous DFT studies of the oxidation of alcohols to aldehydes on metal-based catalysts,^54^ and involves the following elementary steps
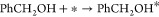
1
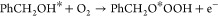
2
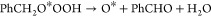
3
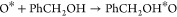
4
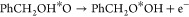
5
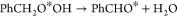
6
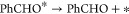
7

By examining the same mechanism and accounting for the presence of oxygen in both Ag_1_@CN_*x*_ catalysts, we observed the formation of identical intermediates. However, as can be seen in the energetic profile (Figure 7c), Ag_1_@CN_*x*_ with a heptazine pore (red line) is more reactive than the new structure of Ag_1_@CN_*x*_ (blue line). The reduced reactivity of the new structure is primarily attributed to the high coordination of the metal site and the limited ability to form new bonds. Notably, throughout all mechanisms, the Ag atom maintains its coordination with the nitrogen atoms (four bonds), except in the case of the second intermediate, where it coordinates with only two nitrogen atoms. On the other side, the Ag atom in the heptazine pore of CN_*x*_ exhibits greater mobility as it changes its coordination with the nitrogen atoms throughout the entire mechanism. Therefore, below we focus on discussing the mechanism involving Ag_1_@CN_*x*_ on the heptazine pore.

The first step was the adsorption of the alcohol on the single metal site (*), to obtain a PhCH_2_OH* intermediate weakly bound (Δ*G* = −0.18 eV) to the Ag atom coordinated to CN_*x*_ (Figure 7c, I). After this step, the oxygen molecule reacts with the intermediate from the first step and an electron transfer process happens to give the PhCH_2_O*OOH intermediate with a barrier of ca. 0.88 eV (Figure 7c, II). The second step is identified as the rate-determining step of the reaction (Figure 7c). Based on this analysis, Ag_1_@CN_*x*_ on the heptazine pore was identified to be the best catalyst, requiring approximately 0.88 eV to overcome this barrier (red line), compared to 1.53 eV with the new structure (blue line). Then, a water molecule and the first benzaldehyde molecules are released, and the process is exergonic with a free energy equal to −2.05 eV, and it leaves O* adsorbed to the Ag-based single-atom catalyst (Figure 7c, III). The oxidation of the second alcohol molecule starts after its adsorption to the catalyst (Figure 7c, IV). The process is slightly exergonic (Δ*G* = −0.29 eV), comparable to the adsorption of the first alcohol molecule (Δ*G* = −0.18 eV). The next step involves the formation of PhCH_2_O*OH intermediate through a second electron transfer process with a free energy of ca. −0.26 eV (Figure 7c, V). The process is followed by the formation of an adsorbed benzaldehyde PhCHO* with the release of a water molecule (Figure 7c, VI). The process is highly exergonic, with a Δ*G* = −2.09 eV. Once the benzaldehyde molecule forms, the catalyst is not able to bind the aldehyde and the oxygenates at the same time, thus blocking the formation of benzoic acid. On the contrary, the catalyst is able to further adsorb and oxidize the alcohol molecule. Therefore, benzyl alcohol acts as an inhibitor, in line with the seminal work of Hutchings and co-workers.^55^ In the last step, the product is desorbed from the single-atom catalyst within an energetic barrier of 0.58 eV (Figure 7c, VII). It should be mentioned that the starting coordination of the catalyst is 4-fold in the heptazine cavity of CN_*x*_. Once the reaction proceeds, the metal atom changes coordination to bind the reaction intermediates. Figure 7c shows the Gibbs free energy profile calculated at *T* = 303 K. The overall reaction is exergonic since the process (2PhCH_2_OH + O_2_ → 2PhCHO + 2H_2_O) has a Δ*G* = −3.41 eV. Specific bond distances for each intermediate with both catalysts are reported in Figures S10 and S11. DFT predicts that this process, involving molecular oxygen, is expected to be thermodynamically more feasible than the same reactions without oxygen (Figure S9 and details in the Supporting Information). In the latter case, in fact, the first step is the adsorption of the reactant on the single metal site (*), to obtain a weakly physisorbed PhCH_2_OH* intermediate (Δ*G* = −0.18 eV). Then, the first proton and electron are released, giving PhCH_2_O* with a barrier of ca. 1.01 eV. A second proton/electron release takes place at this stage, leading to the formation of the adsorbed benzaldehyde (PhCHO*). Finally, the product is desorbed from the single-atom catalyst. The overall reaction (PhCH_2_OH → PhCHO + 2H^+^ + 2e^–^) has a Δ*G*_overall_ = +0.41 eV. However, this alternative profile is expected to be extremely unfavorable because the barrier to form the second reaction intermediate is thermodynamically unfeasible. Therefore, in the absence of oxygen, this intermediate would accumulate on the surface and block the active sites. This result does not change by altering the type of model structure (see the comparison between the blue and black paths in Figure S9).

## 4 Conclusions

In conclusion, we have demonstrated the successful synthesis and application of a silver-based single-atom catalyst (Ag_1_@CN_*x*_) in the photocatalytic oxidation of benzyl alcohol to benzaldehyde. Extensive characterization, including XPS, aberration-corrected electron microscopy, and XAS, confirmed the atomic dispersion of silver on carbon nitride. Catalytic experiments revealed the synergy between silver atoms, molecular oxygen, and visible light in achieving high turnover numbers in the selective oxidation, surpassing all reported protocols driven by visible light. Mechanistic insights obtained from DFT calculations and operando spectroscopy, including transient absorption spectroscopy, revealed that silver atoms play a pivotal role in facilitating and maintaining effective charge separation in Ag_1_@CN_*x*_, significantly enhancing the selective oxidation of benzyl alcohol to benzaldehyde. DFT calculations supported the presence of a radical mechanism, where the interaction between the silver atoms and molecular oxygen was found to be energetically favorable. Overall, this work pioneers the use of Ag-based single-atom catalysts in the photocatalytic oxidation of benzyl alcohol and contributes to the fundamental understanding of charge dynamics in the process.

## Data Availability

All the data supporting the findings of this study are available within the article and its Supporting Information and also from the corresponding authors upon reasonable request.

## Supporting Information Available

It includes additional characterization and catalytic results of the materials; EXAFS fitting results; density of states and additional DFT calculations; additional TAS data; and NMR characterizations of the reaction products (PDF)
