# Supplementary material for: Tracking Charge Dynamics in a Silver Single-Atom Catalyst During the Light-Driven Oxidation of Benzyl Alcohol to Benzaldehyde
Source: ACS Catal. 2025 Mar 21;15(7):5601–13. doi: 10.1021/acscatal.4c05208 (PMC11976699; doi:10.1021/acscatal.4c05208)
Supplement: Supplementary file 1 — cs4c05208_si_001.pdf [file cs4c05208_si_001.pdf]

Supporting Information

Tracking Charge Dynamics in a Silver Single-Atom Catalyst During the Light-Driven Oxidation of Benzyl Alcohol to Benzaldehyde

Areti Moutsiou,[a,†] Andrea Olivati,[b,c,†] Luis A. Cipriano,[a] Alessandra Sivo,[a] Sean M. Collins,[d,e] Quentin M. Ramasse,[e,f] Ik Seon Kwon,[g] Giovanni Di Liberto,[h] Mohamad Kanso,[i] Robert Wojcieszak,[i] Gianfranco Pacchioni,[h] Annamaria Petrozza,[b] and Gianvito Vilé,[a]*

[a] *Department of Chemistry, Materials, and Chemical Engineering "Giulio Natta", Politecnico di Milano, Piazza Leonardo da Vinci 32, 20133 Milano, Italy.*

[b] *Center for Nanoscience and Technology, Italian Institute of Technology, via Giovanni Pascoli 70/3, 20133 Milano, Italy.*

[c] *Physics Department, Politecnico di Milano, Piazza Leonardo da Vinci 32, 20133 Milano, Italy.*

[d] *Bragg Centre for Materials Research, School of Chemical and Process Engineering and School of Chemistry, University of Leeds, Woodhouse Lane, LS2 9JT Leeds, United Kingdom.*

[e] *SuperSTEM Laboratory, SciTech Daresbury Campus, Keckwick Lane, WA4 4AD Daresbury, United Kingdom.*

[f] *School of Chemical and Process Engineering and School of Physics, University of Leeds, Woodhouse Lane, LS2 9JT Leeds, United Kingdom.*

[g] *Department of Energy Science & Engineering, Kunsan National University, 558 Daehak-ro, Gunsan, Republic of Korea.*

[h] *Department of Materials Science, University of Milan Bicocca, via Roberto Cozzi 55, 20125 Milano, Italy.*

[i] *Centre National de la Recherche Scientifique (CNRS) and Laboratoire Lorraine de Chimie Moléculaire, L2CM UMR 7053, Université de Lorraine, 54500 Vandœuvre-lès-Nancy, France.*

† Areti Moutsiou and Andrea Olivati contributed equally to this work.

** Corresponding author. E-mail: gianvito.vile@polimi.it (Gianvito Vilé).*

Table S1. *Elemental composition and textural properties of CN_x and $Ag_1@CN_x$.*

Catalyst	C^a (wt %)	N^a (wt %)	H^a (wt %)	C/N (-)	Ag^b (wt.%)	$S_{BET}{}^c$ ($m^2 g^{-1}$)	$d_{pore}{}^d$ (nm)
CN_x	30.41	52.26	1.80	0.58	-	181	10.78
$Ag_1@CN_x$	31.01	47.43	2.40	0.65	0.30	174	11.33

[a]CHNS; [b]ICP-OES; [c]N_2 isotherm collected at 77 K. [d]BJH pore diameter.

Table S2. *Structural information derived from EXAFS analysis of Ag foil and $Ag_1@CN_x$.*

Sample	Scattering path	N^a	R^b (Å)	$\sigma^{2\,c}$ (10^{-3} Å^2)	$\Delta E_0{}^d$ (eV)	R-factor[e]
Ag foil	Ag-Ag	12	2.861±0.001	9.06±0.20	1.42±0.26	0.001
$Ag_1@CN_x$	$Ag\text{-}N_1$	1	2.015±0.055	9.34±6.74	4.99±1.07	0.014
	$Ag\text{-}N_2$	3	2.197±0.028	7.15±2.36		
	Ag-C	1	2.501±0.071	10.4±6.99		

[a]Coordination number. [b]Interatomic distance. [c]Debye-Waller factor. [d]Edge-energy shift. [e]The fit was carried out in R-space, and the R-factor is the measure of the goodness of fit. The higher the R-factor, the lower the misfit is.

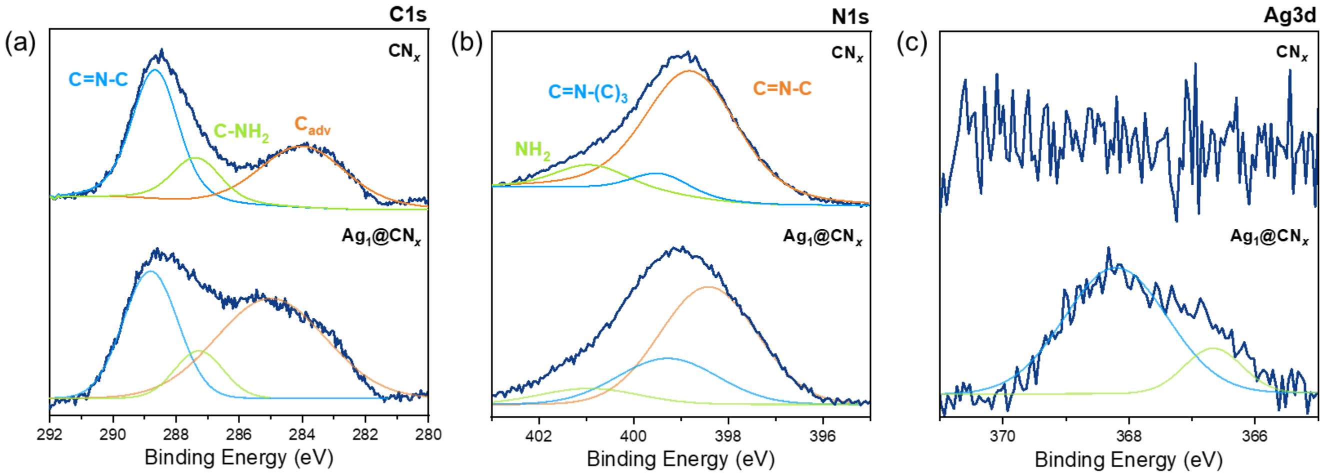

Figure S1. *C 1s (a), N 1s (b), and Ag 3d (c) X-ray photoelectron spectroscopy of CN_x (top) and $Ag_1@CN_x$ (bottom).*

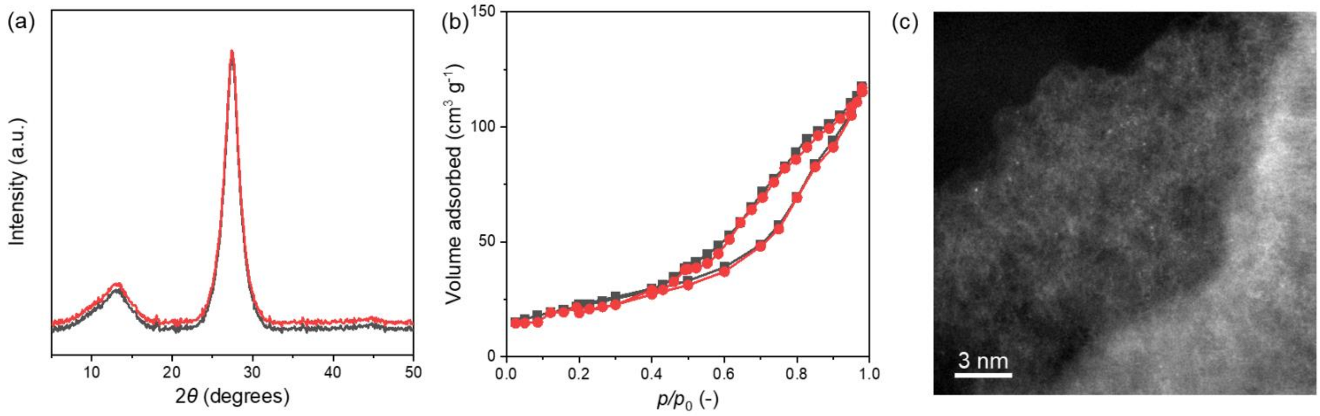

Figure S2. *X-ray diffractograms (a) and N_2 physisorption isotherms (b) of Ag_1@CN_x before (black) and after (red) catalytic tests at 30 °C for 240 min. Atomic-resolved HAADF-STEM micrograph (c) of the Ag_1@CN_x catalyst after reaction, showing no agglomeration of metal species.*

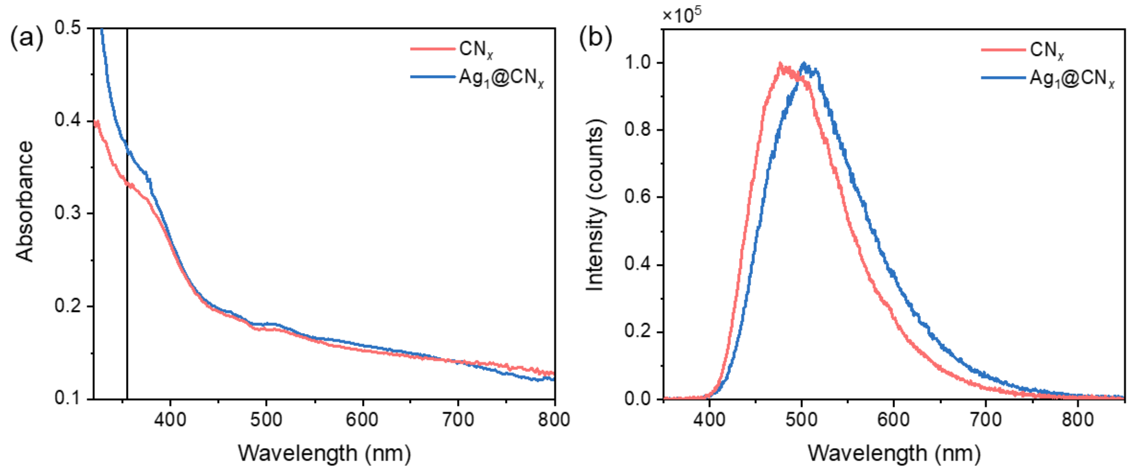

Figure S3. *Absorption spectra of CN_x (red) and Ag_1@CN_x (black) solutions in acetonitrile (a). PL normalized spectra of of CN_x and Ag_1@CN_x (b).*

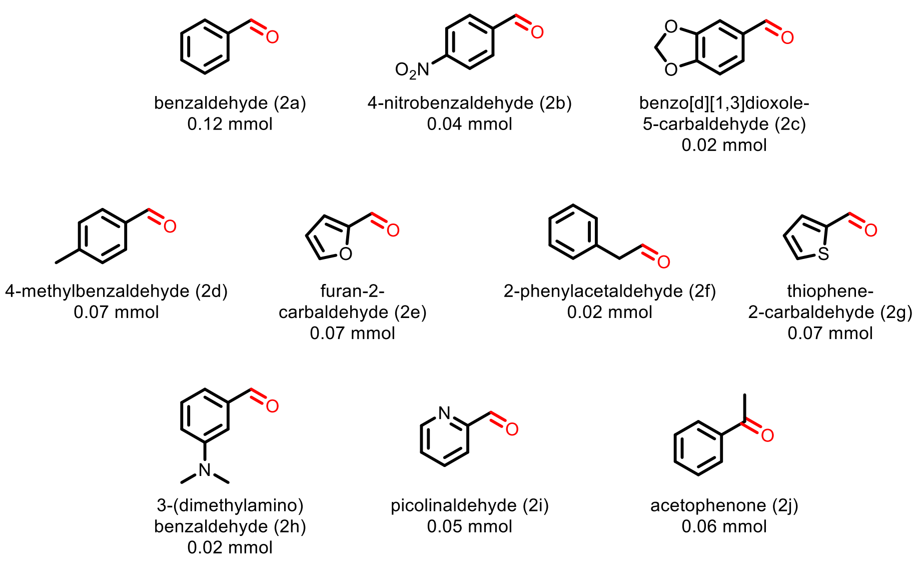

Figure S4. *Substrate scope for the photocatalytic oxidation of alcohols to aldehydes over $Ag_1@CN_x$. Each reaction was performed from the corresponding alcohol using 0.18 mmol of reactant. Experimental conditions and NMR product analysis on page S10, Supporting Information.*

Table S3. *Comparison of catalytic activities for the selective photo-oxidation of benzyl alcohol to benzaldehyde over various catalysts.*

Entry	Catalyst	Reaction conditions	Rate	Ref.
1	$Ag_1@CN_x$	Acetonitrile, 30 °C, 1 h	34.0	This work
2	3Cu-CN	1 bar O_2, acetonitrile, 25 °C, 3 h	0.2	[1]
3	RuSA-RuO_2/TiO_2	Ar, H_2O, 8 °C, 4 h	1.4	[2]
4	Pt/5MnNS	1 bar O_2, benzotrifluoride, 25 °C, 4 h	1.2	[3]
5	(Pt-Au)/TiO_2-MP	1 bar O_2, acetonitrile, 25 °C, 5 h	2.0	[4]
6	C_3N_4/TiO_2(B)	5 bar O_2, acetonitrile, 60 °C, 3 h	1.2	[5]
7	TiO_2/Ti_3C_2	1 bar O_2, hexane, 15 °C, 5 h	0.1	[6]
8	CNNA	1 bar O_2, acetonitrile, 25 °C, 9 h	3.8	[7]
9	$BiVO_4$/g-C_3N_4 (5/5)	1 bar O_2, acetonitrile, 25 °C, 16 h	0.7	[8]
10	0.3rGO–CNS	1 bar O_2, benzotrifluoride, 35 °C, 8 h	1.1	[9]

Rate: $\mu mol_{product}\ mg_{catalyst}^{-1}\ h^{-1}$

Table S4. *Effect of metal loading on benzaldehyde formation.*

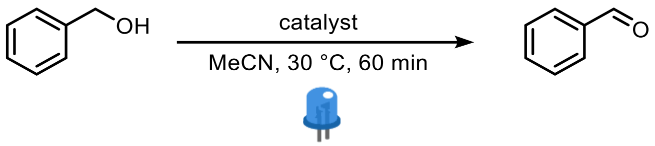

Catalyst	Rate ($\mu mol_{product}\ mg_{catalyst}^{-1}\ h^{-1}$)
$Ag_1@CN_x$	34.0
CN_x	2.1

Reaction conditions: benzyl alcohol concentration = 37 mmol L^{-1}, in MeCN, at 30 °C, for 60 min, using 5 mg of photocatalyst, and blue (450 nm) light. The quantity of benzaldehyde mmol was determined via GC-MS using a calibration curve.

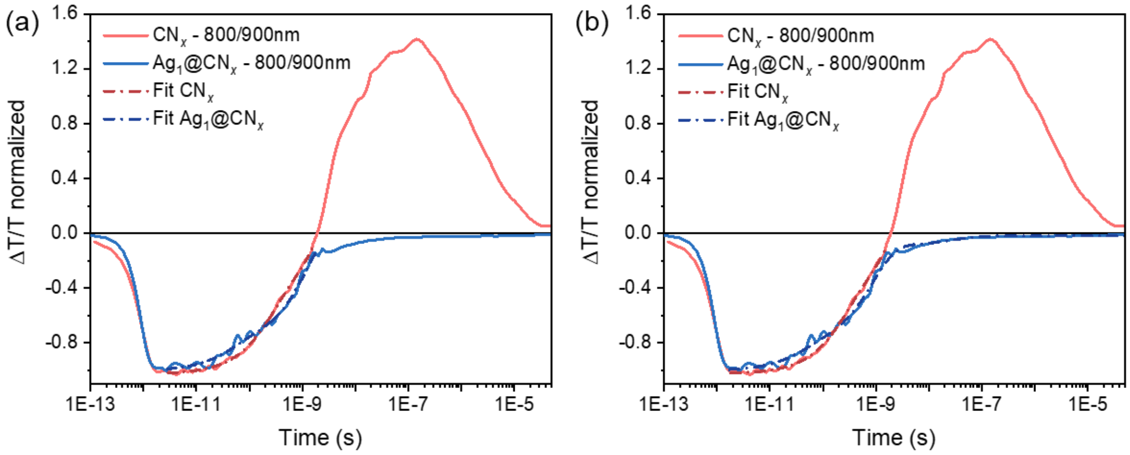

Figure S5. *Two examples of fitting of the photoinduced absorption of CN_x and $Ag_1@CN_x$.*

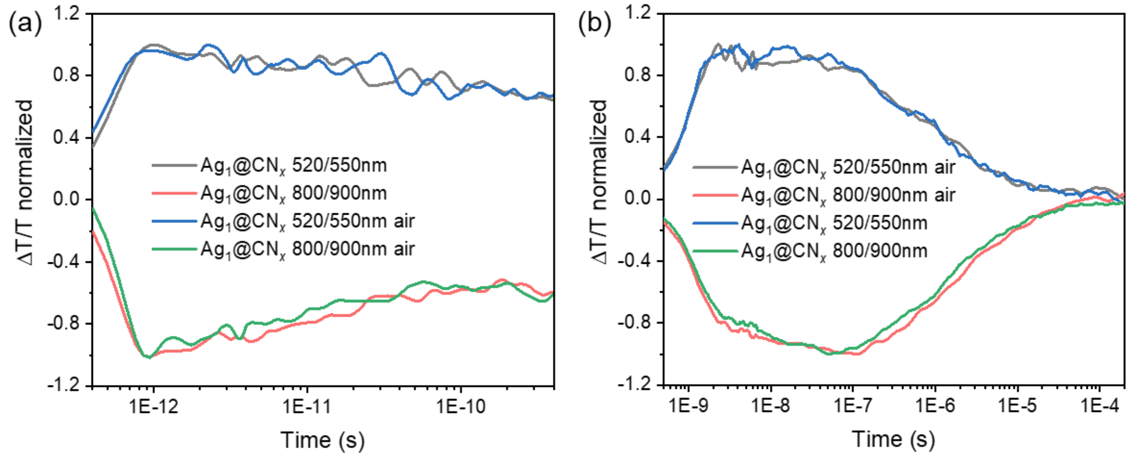

Figure S6. *Comparison of the dynamics collected on $Ag_1@CN_x$ before and after exposure of the solution to air (a), and with and without oxygen in the solvent (b). No appreciable differences can be noted.*

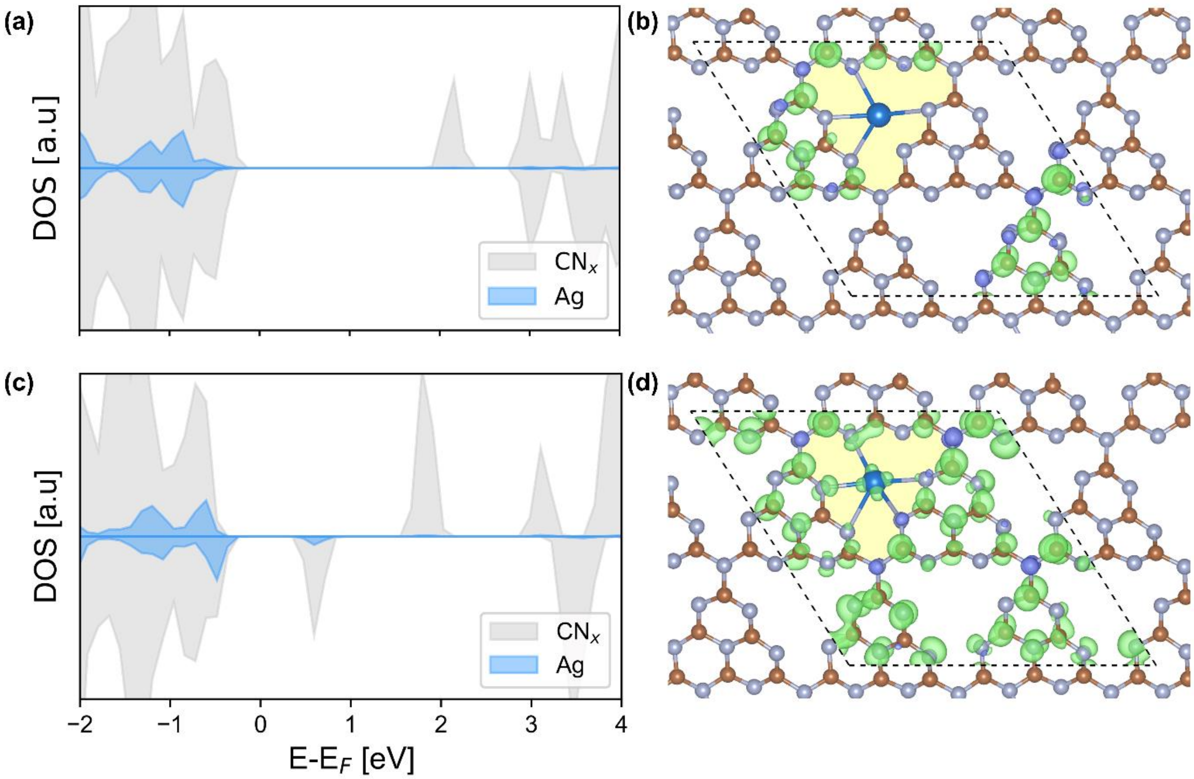

Figure S7. *Density of states (a) and spin density isosurface for the doublet ground state of $Ag_1@CN_x$ (b). Density of states (c) and spin density isosurface of the quartet excited state of $Ag_1@CN_x$ (d). Spin density isosurfaces at 0.003 e/A^3.*

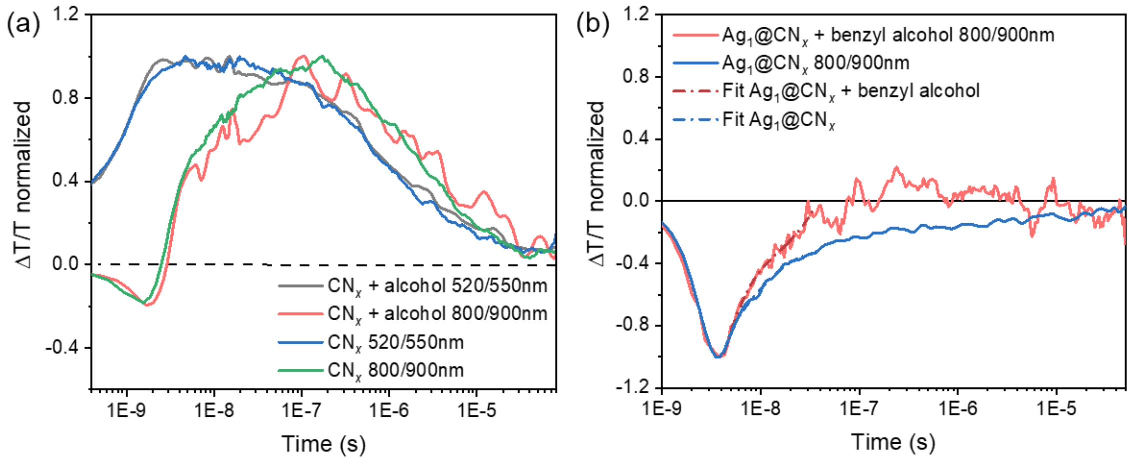

Figure S8. *Comparison of the signal collected from μs-TA measures in the 520/550nm and 800/900nm range on CN_x in acetonitrile with and without the addition of benzyl alcohol (37 mmol L^{-1}). The introduction of the reactant in the system does not affect the dynamics (a). PIA decays of $Ag_1@CN_x$ with and without the addition of benzyl alcohol with the relative fitting curves (b).*

Table S5. *Benzaldehyde formation over $Ag_1@CN_x$ in the presence of different radical scavengers*

Entry	Scavenger[a]	TON (mmol$_{prod}$ mmol$_{Ag}^{-1}$)[b]
1	none	687
2	KI	0
3	CCl_4	684

[a]The reaction was conducted at a benzyl alcohol concentration of 37 mmol L^{-1}, in the presence of 1 equivalent of various radical scavengers, in MeCN, at 30 °C, for 30 min, using $Ag_1@CN_x$ as photocatalyst (5 mg), and blue (450 nm) light. [b]The quantity of benzaldehyde mmol was determined via GC-MS using a calibration curve.

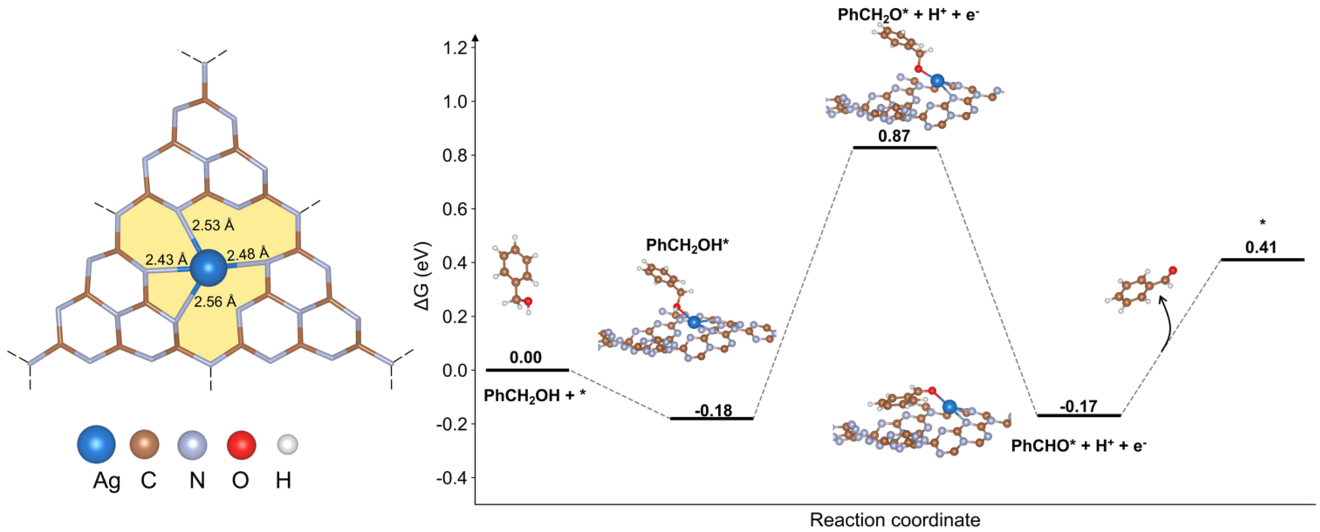

Figure S9. *Calculated Gibbs free energy profile calculated at T = 303 K of the oxidation of benzyl alcohol to benzaldehyde in the absence of oxygen in the catalytic cycle. Brown, blue, light purple, red, and white balls correspond to carbon, silver, nitrogen, oxygen, and hydrogen atoms.*

The model structures of Ag_1@CN_x consider an Ag entrapped in the heptazine pore of CN_x (blue profile). For the catalytic cycle, we considered the following reaction path:

$PhCH_2OH + * \rightarrow PhCH_2OH*$

$PhCH_2OH* \rightarrow PhCH_2O* + H^+ + e^-$

$PhCH_2O* \rightarrow PhCHO* + H^+ + e^-$

$PhCHO* \rightarrow PhCHO + *$

The first step is the adsorption of the reactant on the single metal site (∗), to obtain a chemisorbed $PhCH_2OH*$ intermediate (ΔG = -0.18 eV). Then, the first proton and electron are released, giving $PhCH_2O*$ with a barrier of ca. 1.01 eV. A second proton/electron release takes place at this stage, leading to the formation of the adsorbed benzaldehyde (PhCHO). Finally, the product is desorbed from the single-atom catalyst. The overall reaction ($PhCH_2OH \rightarrow PhCHO + 2H^+ + 2e^-$) has a $\Delta G_{overall}$ = +0.41 eV. However, a reaction in the absence of oxygen is expected to be extremely unfavorable because the barrier to form the second reaction intermediate is extremely high. This result does not change by altering the type of model structure (see the figure above, comparing the blue and black paths).*

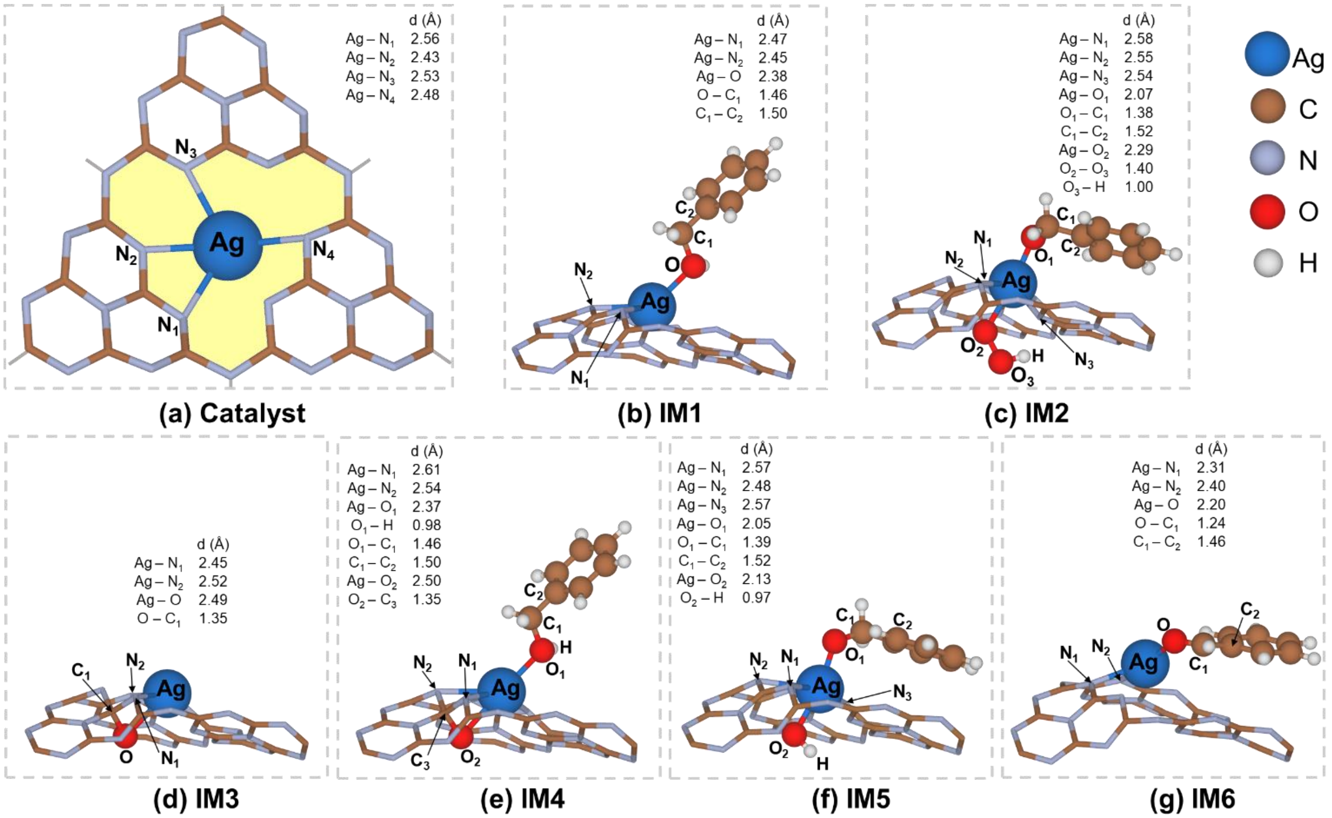

Figure S10. *Bond distances for the a) catalyst and b-g) each intermediate (IM) of the oxidation of benzyl alcohol to benzaldehyde on $Ag_1@CN_x$ with a heptazine pore. b) IM1 corresponds to the intermediate formed from step I, c) IM2 from step II, d) IM3 from step III, e) IM4 from step IV, f) IM5 from step V and g) IM6 from step VI, respectively. Color codes: brown = carbon, blue = silver, light purple = nitrogen, red = oxygen, and white = hydrogen.*

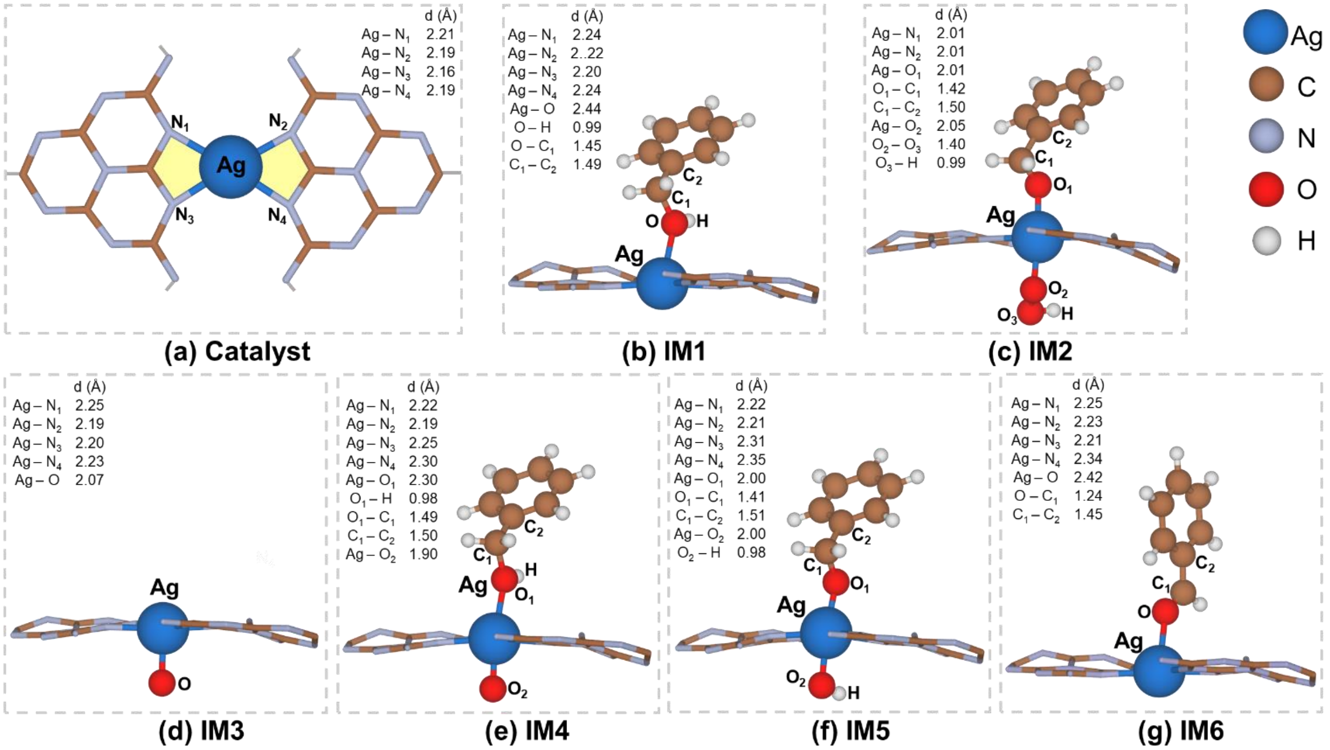

Figure S11. *Bond distances for the a) catalyst and b-g) each intermediate (IM) of the oxidation of benzyl alcohol to benzaldehyde on the new structure of Ag_1@CN_x. b) IM1 corresponds to the intermediate formed from step I, c) IM2 from step II, d) IM3 from step III, e) IM4 from step IV, f) IM5 from step V and g) IM6 from step VI, respectively. Color codes: brown = carbon, blue = silver, light purple = nitrogen, red = oxygen, and white = hydrogen.*

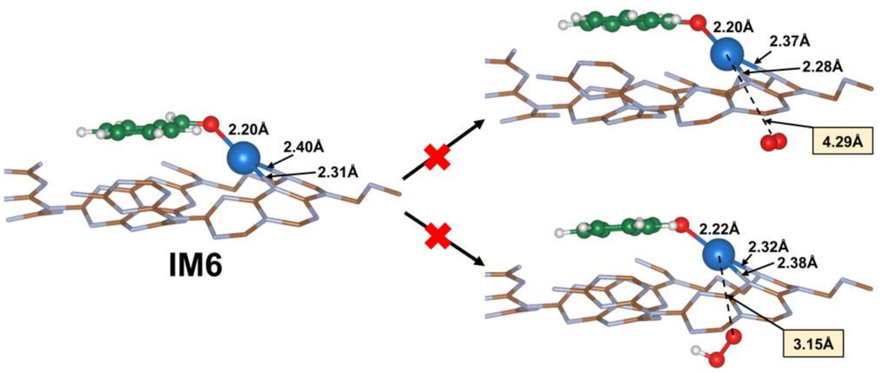

Figure S12. *Side view of benzaldehyde adsorbed on Ag_1@CN_x (IM6, left side) and side view of benzaldehyde and the not adsorbed oxygenates on Ag_1@CN_x (right side).*

Reaction conditions and NMR characterization of the compounds in Figure S3

General procedure. The photocatalytic oxidation of various alcohols was performed using an Illumin8 parallel photoreactor (Asynt, UK), equipped with DrySyn OCTO MINI 8-position reaction station, standard hotplate magnetic stirring, and a cooling system to control the reaction temperature. In a typical procedure, the alcohol (0.18 mmol) was mixed with 5 mL of solvent (acetonitrile) and 5 mg of catalyst ($Ag_1@CN_x$). The reaction flask was irradiated with blue light (λ = 450 nm, with 10 W LED COB chips) under continuous stirring (400 rpm) for the whole reaction time. All reactions were performed at 30 °C. Reaction products were analyzed *via* gas chromatography (GC) equipped with a flame ionization-mass spectroscopy detector. The detection was done using two columns, HP-INNOWAX (5 m × 0.250 mm × 0.15 μm) and DB-5MS (20 m × 0.180 mm × 0.18 μm), connected in series. Quantification was performed after calibration of the GC with a standard.

benzaldehyde (2a)

Prepared according to the general procedure, using benzyl alcohol (19.5 mg, 0.18 mmol). The reaction was carried out for 4 h. **^{1}H NMR (400 MHz, CD_3CN)**: δ 10.04 (s, 1H), 7.95 – 7.90 (m, 2H), 7.74 – 7.68 (m, 1H), 7.64 – 7.58 (m, 2H).

4-nitrobenzaldehyde (2b)

Prepared according to the general procedure, using (4-nitrophenyl)methanol (27.6 mg, 0.18 mmol). The reaction was carried out for 6 h. **^{1}H NMR (400 MHz, CD_3CN)**: δ 10.10 (s, 1H), 8.34 (d, J = 8.7 Hz, 2H), 8.07 (d, J = 8.8 Hz, 2H).

benzo[d][1,3]dioxole-5-carbaldehyde (2c)

Prepared according to the general procedure, using benzo[d][1,3]dioxol-5-ylmethanol (27.4 mg, 0.18 mmol). The reaction was carried out for 6 h. **^{1}H NMR (400 MHz, CD_3CN)**: δ 9.82 (s, 1H), 7.49 (dd, J = 8.0, 1.6 Hz, 1H), 7.32 (d, J = 1.6 Hz, 1H), 7.02 (d, J = 7.9 Hz, 1H), 6.11 (s, 2H).

4-methylbenzaldehyde (2d)

Prepared according to the general procedure, using p-tolylmethanol (22.0 mg, 0.18 mmol). The reaction was carried out for 6 h. **^{1}H NMR (400 MHz, CD_3CN)**: δ 9.97 (s, 1H), 7.81 (d, J = 8.1 Hz, 2H), 7.42 (d, J = 7.8 Hz, 2H), 2.46 (s, 3H).

furan-2-carbaldehyde (2e)

Prepared according to the general procedure, using furan-2-ylmethanol (16.6 mg, 0.18 mmol). The reaction was carried out for 12 h. **^{1}H NMR (400 MHz, CD_3CN)**: δ 9.63 (d, J = 0.8 Hz, 1H), 7.87 – 7.81 (m, 1H), 7.38 (dd, J = 3.6, 0.8 Hz, 1H), 6.70 (dd, J = 3.6, 1.7 Hz, 1H).

2-phenylacetaldehyde (2f)

Prepared according to the general procedure, using 2-phenylethan-1-ol (22.0 mg, 0.18 mmol). The reaction was carried out for 14 h. **^{1}H NMR (400 MHz, CD_3CN)**: δ 10.04 (s, 1H), 7.76 – 7.69 (m, 1H), 7.64 – 7.59 (m, 2H), 7.43 – 7.35 (m, 2H), 3.71 (s, 2H).

thiophene-2-carbaldehyde (2g)

Prepared according to the general procedure, using thiophen-2-ylmethanol (20.5 mg, 0.18 mmol). The reaction was carried out for 6 h. **^{1}H NMR (400 MHz, CD_3CN)**: δ 9.96 (d, J = 1.3 Hz, 1H), 8.12 – 7.83 (m, 1H), 7.46 – 7.19 (m, 1H), 7.00 (s, 1H).

3-(dimethylamino)benzaldehyde (2h)

Prepared according to the general procedure, using 3-(dimethylamino)phenylmethanol (27.2 mg, 0.18 mmol). The reaction was carried out for 6 h. **^{1}H NMR (400 MHz, CD_3CN)**: δ 8.50 (s, 1H), 7.41 (t, J = 7.7 Hz, 1H), 6.61 (d, J = 7.3 Hz, 2H), 6.53 – 6.47 (m, 1H), 3.08 (s, 6H).

picolinaldehyde (2i)

Prepared according to the general procedure, using pyridin-2-ylmethanol (19.6 mg, 0.18 mmol). The reaction was carried out for 6 h. **^{1}H NMR (400 MHz, CD_3CN)**: δ 10.04 (d, J = 0.8 Hz, 1H), 8.82 (d, J = 4.8 Hz, 1H), 8.03 – 7.92 (m, 2H), 7.68 – 7.59 (m, 1H).

acetophenone (2j)

Prepared according to the general procedure, using 1-phenylethan-1-ol (22.0 mg, 0.18 mmol). The reaction was carried out for 6 h. **^{1}H NMR (400 MHz, CD_3CN)**: δ 8.04 – 7.95 (m, 2H), 7.69 – 7.58 (m, 1H), 7.58 – 7.50 (m, 2H), 2.60 (s, 3H).

References of the Supporting Information

[1] Sudrajat, H.; Wella, S. A.; Phanthuwongpakdee, J.; Lisovytskiy, D.; Sobczak, K.; Colmenares, J. C. Atomistic Understanding of Enhanced Selectivity in Photocatalytic Oxidation of Benzyl Alcohol to Benzaldehyde Using Graphitic Carbon Nitride Loaded with Single Copper Atoms. *Nanoscale* **2024**, *16* (31), 14813–14830.

[2] Xing, B.; Wang, T.; Zheng, Z.; Liu, S.; Mao, J.; Li, C.; Li, B. Synchronous Fabrication of Ru Single Atoms and RuO_2 on Hierarchical TiO_2 Spheres for Enhanced Photocatalytic Coproduction of H_2 and Benzaldehyde. *Chem. Eng. J.* **2023**, *461*, 141871.

[3] Shi, Y.; Shen, M.; Wang, Z.; Liu, C.; Bi, J.; Wu, L. Visible-Light-Driven Benzyl Alcohol Oxidation over Pt/Mn-$Bi_4Ti_3O_{12}$ Nanosheets: Structure-Function Relationship of Multicomponent Photocatalysts. *J. Catal.* **2023**, *418*, 141–150.

[4] Sun, N.; Song, J.; Tao, Q.; Kan, E.; Kuai, L. High-Loading Single-Atom Pt/TiO_2 Mesoporous Catalysts for Superior Photocatalytic Oxidation of Benzyl Alcohol. *Microporous Mesoporous Mater.* **2022**, *337*, 111949.

[5] Shi, Q.; Zhang, X.; Liu, X.; Xu, L.; Liu, B.; Zhang, J.; Xu, H.; Han, Z.; Li, G. In-Situ Exfoliation and Assembly of 2D/2D g-C_3N_4/TiO_2(B) Hierarchical Microflower: Enhanced Photo-Oxidation of Benzyl Alcohol under Visible Light. *Carbon* **2022**, *196*, 401–409.

[6] Bao, X.; Li, H.; Wang, Z.; Tong, F.; Liu, M.; Zheng, Z.; Wang, P.; Cheng, H.; Liu, Y.; Dai, Y.; Fan, Y.; Li, Z.; Huang, B. TiO_2/Ti_3C_2 as an Efficient Photocatalyst for Selective Oxidation of Benzyl Alcohol to Benzaldehyde. *Appl. Catal. B* **2021**, *286*, 119885.

[7] Ding, J.; Xu, W.; Wan, H.; Yuan, D.; Chen, C.; Wang, L.; Guan, G.; Dai, W.-L. Nitrogen Vacancy Engineered Graphitic C_3N_4-Based Polymers for Photocatalytic Oxidation of Aromatic Alcohols to Aldehydes. *Appl. Catal. B* **2018**, 221, 626–634.

[8] Samanta, S.; Khilari, S.; Pradhan, D.; Srivastava, R. An Efficient, Visible Light Driven, Selective Oxidation of Aromatic Alcohols and Amines with O_2 Using $BiVO_4$/g-C_3N_4 Nanocomposite: A Systematic and Comprehensive Study toward the Development of a Photocatalytic Process. *ACS Sustain. Chem. Eng.* **2017**, *5* (3), 2562–2577.

[9] Xu, J.; Luo, L.; Xiao, G.; Zhang, Z.; Lin, H.; Wang, X.; Long, J. Layered $C_3N_3S_3$ Polymer/Graphene Hybrids as Metal-Free Catalysts for Selective Photocatalytic Oxidation of Benzylic Alcohols under Visible Light. *ACS Catal.* **2014**, *4* (9), 3302–3306.
